# Decoupling Electron and Proton Transfer in Noncontact Catalytic Hydrogenation of Nitroaromatics

**DOI:** 10.1002/advs.202511391

**Published:** 2025-08-28

**Authors:** Zhongyin Liang, Qingyuan Wu, Zhe Yang, Nanfeng Zheng

**Affiliations:** ^1^ New Cornerstone Science Laboratory State Key Laboratory for Physical Chemistry of Solid Surfaces Collaborative Innovation Center of Chemistry for Energy Materials and National & Local Joint Engineering Research Center for Preparation Technology of Nanomaterials College of Chemistry and Chemical Engineering Xiamen University Xiamen 361005 China; ^2^ Innovation Laboratory for Sciences Technologies of Energy Materials of Fujian Province (IKKEM) Xiamen 361102 China

**Keywords:** catalytic mechanism, conductive carbon support, electron transfer, nitroaromatics hydrogenation, noncontact catalysis, proton transfer

## Abstract

Understanding the elementary reactions of active hydrogen species and electron transfer mechanisms during catalytic hydrogenation remains a fundamental challenge. This work elucidates electron and proton transfer pathways in nitroaromatics hydrogenation using a noncontact catalytic system. Strong coordination of (hypo)phosphorous acid on Pt/Pd surfaces prevents substrate adsorption while permitting H_2_ activation, generating electrons retained on the catalyst and protons solvated in protic solvents. Hydrogenation proceeds via sequential reduction (nitro to nitroso and then to hydroxylamine), governed primarily by electron transfer through conductive carbon supports and secondarily via catalyst interfaces, while proton transfer occurs through protic solvents. Disproportionation dominates hydroxylamine conversion due to its kinetic superiority over direct hydrogenation. By applying these mechanistic insights, the system is expanded to diverse catalysts, demonstrating that hypophosphorous acid‐modified commercial Pt/C catalyst achieves efficient nitroaromatics hydrogenation. Remarkably, this approach functionally mimics enzymatic catalysis, enabling selective hydrogenation of coenzyme Q10 and its analogues. This study advances fundamental understanding of hydrogenation mechanisms of nitroaromatics, carbon‐supported catalyst design, and enzyme‐mimetic catalysis development.

## Graphical abstract

A noncontact catalytic system is constructed by modifying metal nanoparticles supported on conductive carbon with (hypo)phosphorous acid. This design effectively prevents the adsorption of unsaturated substrates while permitting H_2_ activation to generate electrons and protons. The hydrogenation is governed by electron transfer processes, which occur at carbon support surfaces and ligand/support‐metal interfaces, with protons transferred via protic solvents.

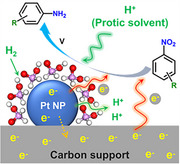

## 1 Introduction

**Scheme 1:**
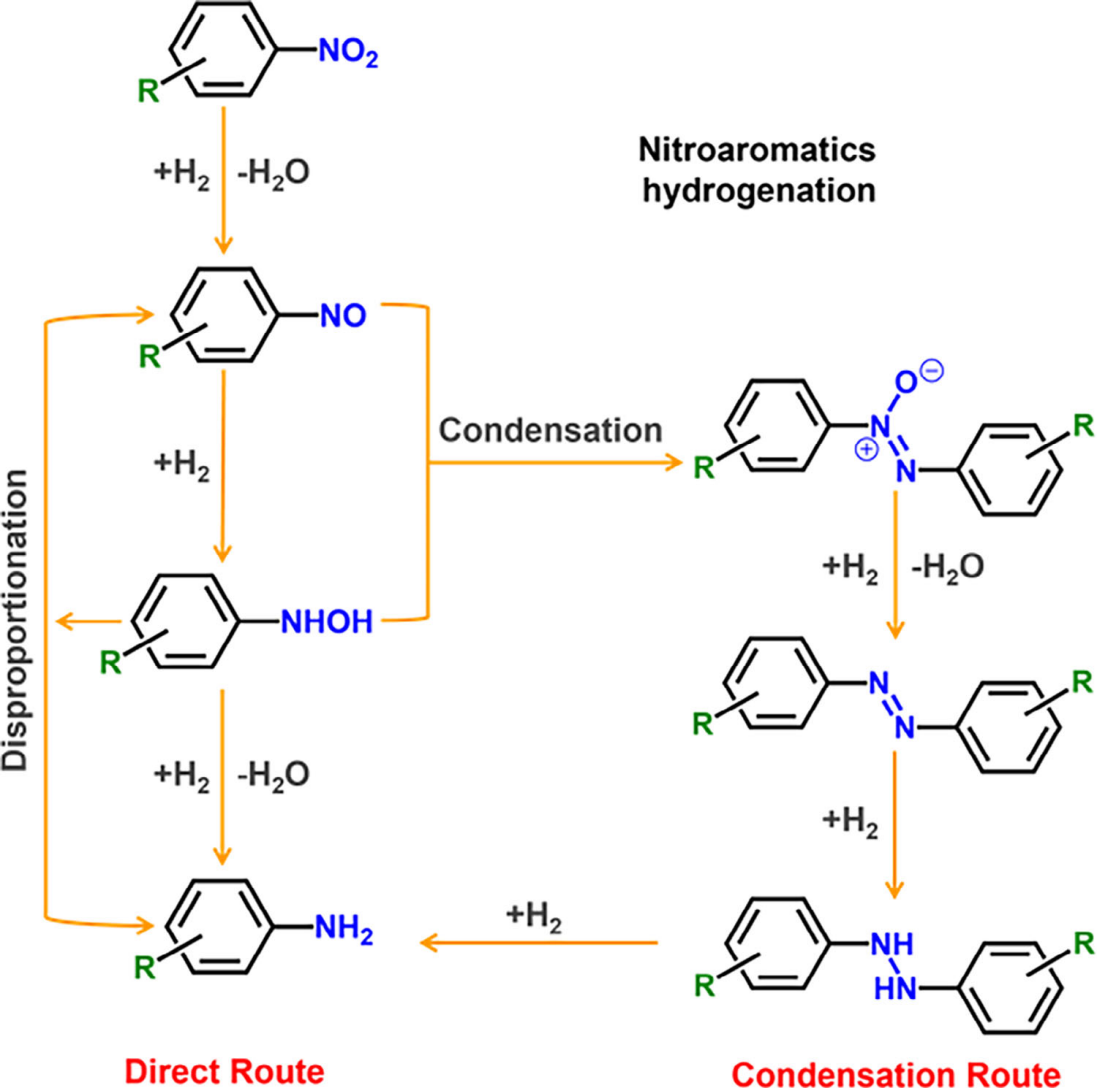
Reaction network of the hydrogenation of nitroaromatics to amines. R: reducible substituents.

Catalytic hydrogenation is of significant industrial and scientific importance due to its broad applications in the production of numerous fine chemicals, such as pharmaceuticals, dyes, food additives, and perfumes.^[^ ^1^, ^2^, ^3^ ^]^ According to the Horiuti–Polanyi mechanism, hydrogenation occurs on the active metal surface:^[^ ^4^, ^5^ ^]^ H_2_ first binds to an active metal atom (such as Pt, Pd, Ni) via its σ‐bond (agostic bonding), and then undergoes a low‐energy H─H bond activation. The unsaturated substrates subsequently adsorb onto the metal surface, where they react with the active hydrogen species to produce products. However, poor catalytic selectivity often arises due to the complex structure of active sites, unpredictable binding modes, and varying adsorption strengths of substrates/intermediates.^[^ ^6^, ^7^, ^8^ ^]^ This challenge is exacerbated in reactions involving competitive pathways, which can generate multiple intermediates or undesired products.^[^ ^9^ ^]^ A notable example is the selective hydrogenation of nitroaromatics to amines, an important industrial process.^[^ ^10^, ^11^, ^12^, ^13^ ^]^ This transformation occurs via direct and condensation routes, often competing with the reduction of other functional groups (**Scheme** **1**).^[^ ^14^, ^15^ ^]^

The selectivity of catalytic hydrogenation can be optimized by engineering active sites to control reactant interaction energies and configurations.^[^ ^16^ ^]^ In heterogeneous catalysis, supports critically influence catalytic performance by modulating the geometric and electronic structure of the active metal phase.^[^ ^17^, ^18^, ^19^, ^20^, ^21^, ^22^ ^]^ Among the most widely used supports are conductive carbon materials, such as activated carbons, carbon blacks, graphitic materials, carbon nanotubes, and carbon nanofibers.^[^ ^23^, ^24^ ^]^ Precious metals supported on carbon provide exceptional versatility in tailoring catalytic properties for fine‐chemical synthesis,^[^ ^25^, ^26^ ^]^ owing to their high thermal stability under reducing conditions and their ability to stabilize well‐dispersed metal nanoparticles.^[^ ^23^, ^27^ ^]^ However, the complex and poorly understood chemistry of carbon materials poses a persistent challenge in establishing a clear structure‐activity relationship, despite growing research efforts to elucidate the behavior of carbon‐supported catalysts.^[^ ^23^, ^28^ ^]^

In this study, we developed a noncontact catalytic hydrogenation system to systematically investigate the critical function of carbon supports in the catalytic mechanism. This approach builds upon our prior research in heterogeneous catalysis while incorporating innovative design elements from recent work employing H‐cell configurations to spatially separate H_2_ activation from substrate hydrogenation.^[^ ^3^, ^29^, ^30^, ^31^ ^]^ A structurally well‐defined Pt/C catalyst consisting of surface‐clean, monodisperse Pt nanoparticles supported on a conductive carbon support was first developed. A critical innovation involves the construction of an isolation layer between the Pt metal surface and the unsaturated substrates using (hypo)phosphorous acid, which effectively prevents direct substrate‐metal contact while allowing selective penetration and activation of H_2_. The activation process generates electrons and protons, which are retained within the catalyst framework and transferred into the protic solvent phase, respectively. Detailed investigation of nitroaromatics hydrogenation reveals distinct reaction characteristics. The conversion of hydroxylamine to amine proceeds significantly more efficiently through disproportionation rather than direct hydrogenation, with the disproportionation rate exceeding the hydrogenation rate by ≈19‐fold. The hydrogenation of nitroso compounds to hydroxylamine occurs very rapidly, exhibiting a reaction rate ≈67 times greater than that of the conversion of nitro into nitroso. This rapid transformation is principally mediated by electron transfer processes occurring predominantly across the conductive carbon support surfaces, with secondary contributions from ligand‐metal interfaces and support‐metal interfaces, together with proton transfer through the protic solvents. The hydrogenation of nitro groups to nitroso intermediates emerges as the rate‐determining step (RDS) in the overall reaction, which is also dominated by electron transfer. The electrons and protons are also transferred through carbon support and protic solvents, respectively. Based on the mechanistic insights, the developed catalytic system is readily expanded by incorporating various metal components, diverse carbon support materials, and different ligands. Furthermore, we achieved selective hydrogenation of biologically significant compounds, including coenzyme Q10 and its analogues, thereby establishing an artificial catalytic system that functionally mimics enzymatic activity.

## 2 Results and Discussion

### 2.1 Synthesis, Characterization, and Catalysis

**Figure 1:**
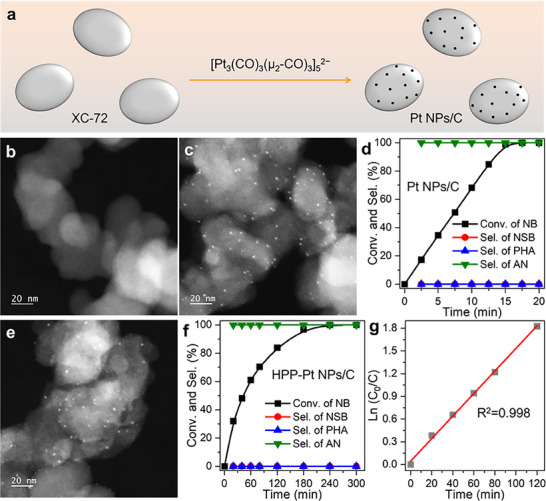
a) Illustration for the synthesis of Pt NPs/C. Representative STEM images of XC‐72 b) and Pt NPs/C c). d) Time‐dependent catalysis of NB hydrogenation to AN on Pt NPs/C. e) Representative STEM image of HPP‐Pt NPs/C. f) Time‐dependent catalysis of NB hydrogenation to AN on HPP‐Pt NPs/C. g) The pseudo‐first‐ order kinetic relationship for NB hydrogenation to AN on HPP‐Pt NPs/C.

The activated carbon (XC‐72) supported monodisperse Pt nanoparticles catalyst (denoted as Pt NPs/C) was first prepared via a surfactant‐free strategy, involving the thermal deposition of [Pt_3_(CO)_3_(μ_2_‐CO)_3_]_5_^2−^ clusters onto carbon supports (**Figure** **1**a).^[^ ^32^ ^]^ As characterized by scanning transmission electron microscopy (STEM, Figure 1b,c; Figures S1 and S2, Supporting Information), transmission electron microscopy (TEM, Figures S1 and S2, Supporting Information) and X‐ray diffraction (XRD, Figure S3, Supporting Information), the as‐prepared Pt NPs/C catalyst showed the good dispersion of uniform and small Pt NPs on XC‐72. The hydrogenation of nitrobenzene (NB) was chosen to evaluate the catalytic performance of the Pt NPs/C catalyst. The reaction was conducted in a glass pressure vessel containing Pt NPs/C (0.002 mmol Pt), NB (1 mmol), vanadium(IV)oxy acetylacetonate [VO(acac)_2_, 0.01 mmol], and ethanol (EtOH, 10 mL) under H_2_ (0.3 MPa) at 60 °C. As evidenced by ^1^H NMR results obtained under oxygen‐isolated conditions (Figure S4, Supporting Information),^[^ ^10^ ^]^ NB was readily hydrogenated to aniline (AN) with a conversion of >99.9% within 15 min (Figure 1d). Neither nitrosobenzene (NSB) nor phenylhydroxylamine (PHA) was detected. Notably, the reaction process follows zero‐order kinetics, as the reaction rate is independent of reactant concentrations (Figure S5, Supporting Information).

When hypophosphorous acid (H_3_PO_2_) was used as a ligand to modify Pt NPs/C (Figure 1e; Figure S6, Supporting Information), the resulting HPP‐Pt NPs/C catalyst achieved the selective hydrogenation of NB to AN within 240 min (Figure 1f; Figure S7, Supporting Information). No obvious decay in conversion or selectivity was observed after six catalytic cycles, demonstrating the robustness of the HPP‐Pt NPs/C catalyst (Figures S8 and S9, Supporting Information). TEM images and XRD patterns revealed no detectable size changes of Pt NPs after the reaction, further confirming the high stability of the catalyst (Figures S10 and S11, Supporting Information). Interestingly, the reaction exhibited pseudo‐first‐order kinetics (Figure 1g),^[^ ^22^ ^]^ in stark contrast to the zero‐order kinetics over Pt NPs/C. This remarkable difference in reaction order suggests distinct active sites and hydrogen mechanisms between the two catalysts. Given that the hydrogenation proceeds on Pt metal surfaces over the unmodified Pt NPs/C catalyst, we propose that the hydrogenation reaction on HPP‐Pt NPs/C does not occur on the exposed metal surface.

### 2.2 Hydrogenation of Nitrobenzene via Noncontact Reaction

**Figure 2:**
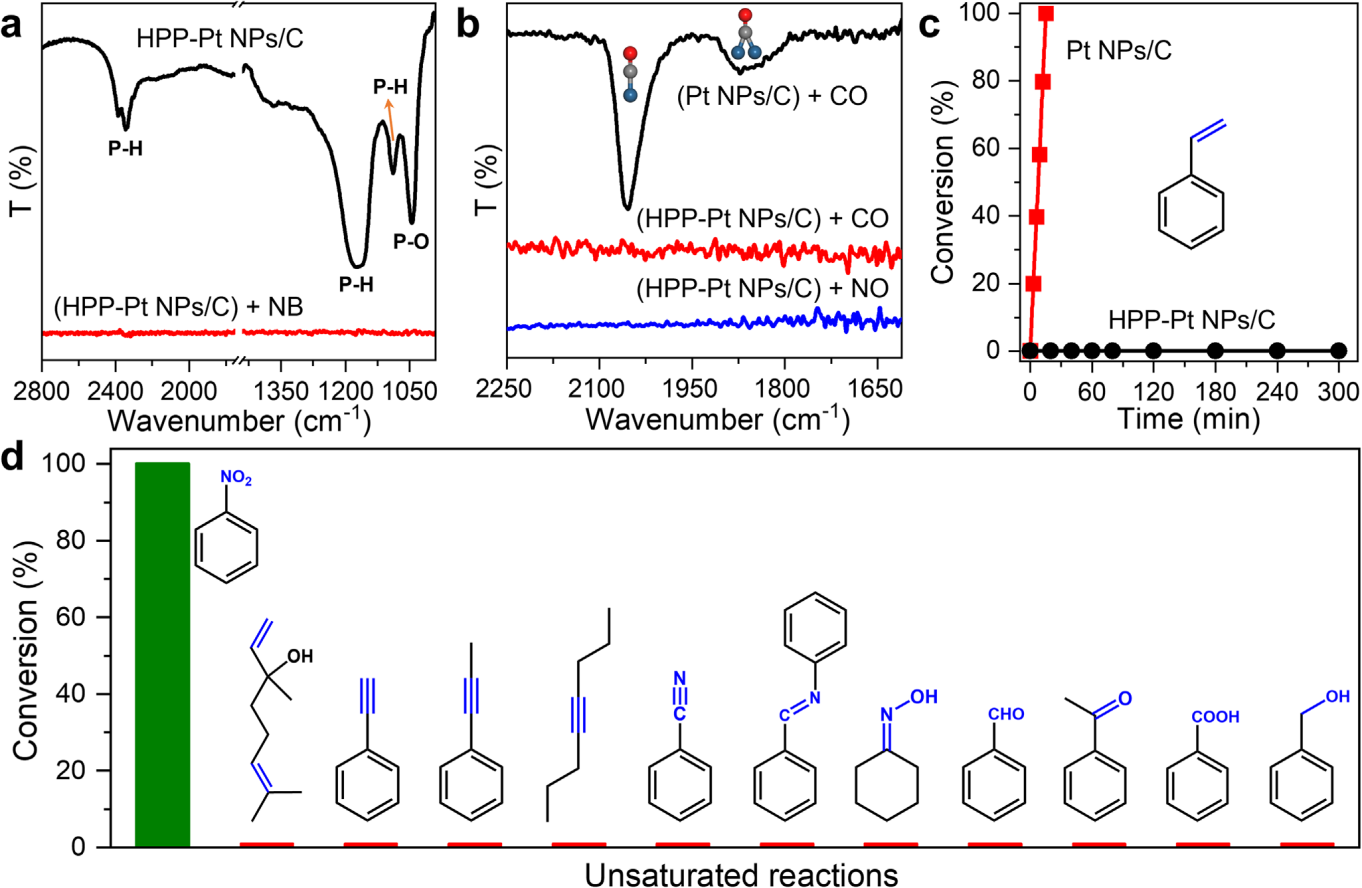
a) In situ DRIFTS spectra of H_3_PO_2_ adsorption on Pt NPs/C and NB adsorption on HPP‐Pt NPs/C. The observed bands at ≈2348, 1168, 1089, and 1045 cm^−1^ were assigned to the P─H symmetric stretching vibrations, P─H rocking vibrations, P─H wagging vibrations, and P─O symmetric stretching vibrations, respectively. b) In situ DRIFTS spectra of CO adsorption on Pt NPs/C and HPP‐Pt NPs/C, as well as NO adsorption on HPP‐Pt NPs/C. c) Catalytic performance of Pt NPs/C and HPP‐Pt NPs/C for styrene hydrogenation. d) Catalytic performance of HPP‐Pt NPs/C for the hydrogenation of NB and other unsaturated compounds. Reaction time: 300 min.

To validate the proposed hypothesis, we first investigated the adsorption behavior of ligands and substrates (e.g., H_3_PO_2_ and NB) was first using in situ diffuse reflectance infrared Fourier transform spectroscopy (DRIFTS). As shown in **Figure** **2**a, H_3_PO_2_ exhibited strong adsorption on Pt NPs/C,^[^ ^33^, ^34^ ^]^ while NB adsorption became negligible when Pt NPs/C was modified with H_3_PO_2_.^[^ ^35^, ^36^ ^]^ This observation was further corroborated by the prominent CO adsorption on bare Pt NPs/C, which was absent on HPP‐Pt NPs/C (Figure 2b). To probe the catalytic surface accessibility, styrene, a molecule easily hydrogenated on exposed Pt surfaces, was employed as a probe reactant.^[^ ^19^, ^37^ ^]^ As expected, Pt NPs/C catalyzed the rapid hydrogenation of styrene (≈1 mmol) within 15 min (Figure 2c; Figure S12, Supporting Information). In sharp contrast, HPP‐Pt NPs/C showed negligible conversion even after 300 min (Figure 2c; Figure S13, Supporting Information), demonstrating that the coordination of H_3_PO_2_ on Pt NPs/C completely blocks adsorption and catalytic hydrogenation of NB. Consistent with these findings, hydrogenation of various unsaturated compounds, including those with C═C, C≡C, C≡N, C═N, C═O, and C─O bonds, was completely suppressed on HPP‐Pt NPs/C (Figure 2d; Figures S14–S16, Supporting Information). Together, these results demonstrated that NB hydrogenation proceeded via a noncontact mechanism over HPP‐Pt NPs/C.

### 2.3 Activation of H_2_ into Protons and Electrons

**Figure 3:**
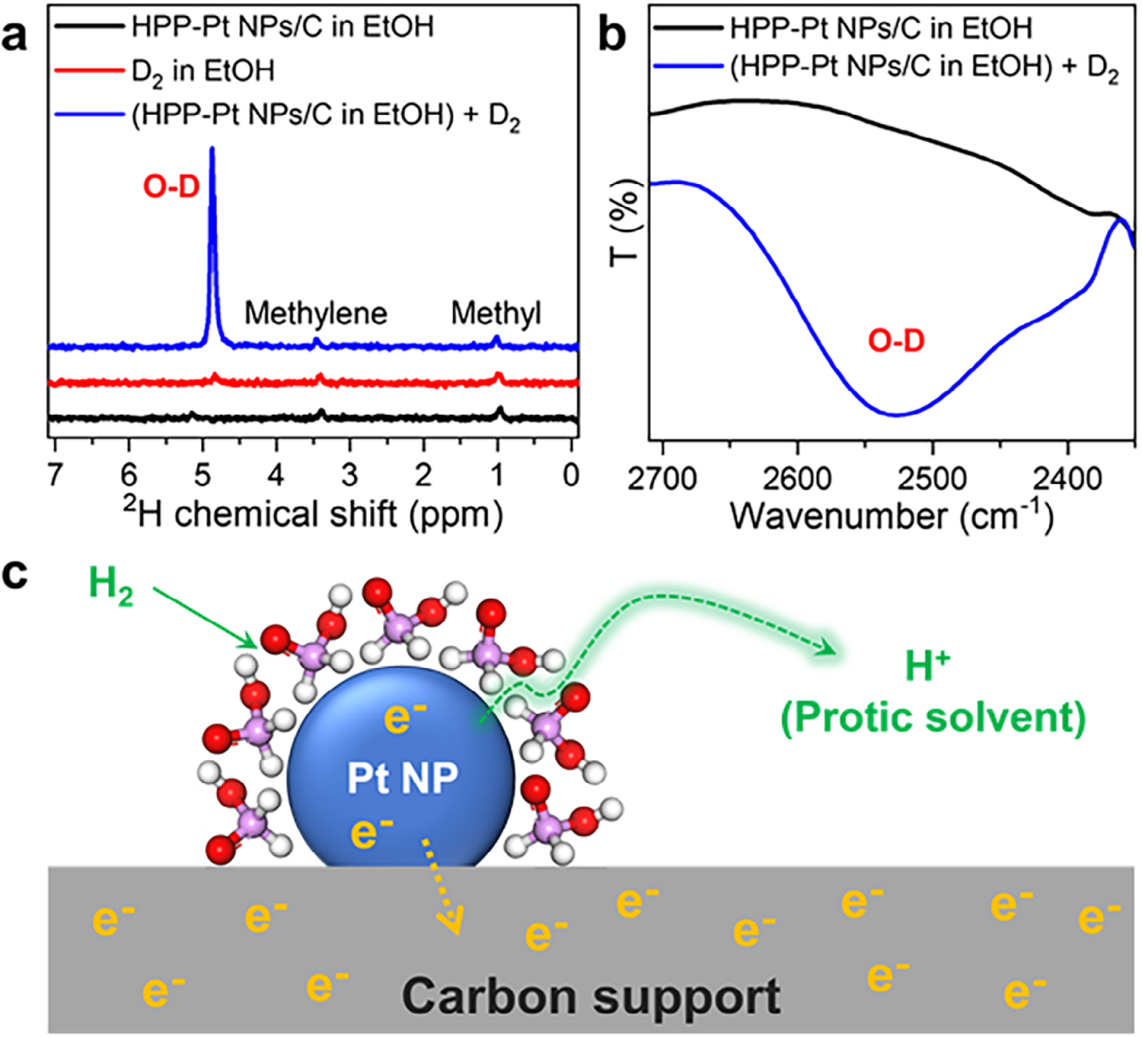
a) ^2^H NMR spectra of HPP‐Pt NPs/C in EtOH, D_2_ in EtOH, and HPP‐Pt NPs/C in EtOH after D_2_ bubbling. b) FTIR spectra of HPP‐Pt NPs/C in EtOH and HPP‐Pt NPs/C in EtOH after bubbling D_2_. c) Illustration of the noncontact catalytic system and the hydrogen activation process. The white, red, and purple balls represent H, O, and P, respectively.

To elucidate how H_2_ was activated in this noncontact catalytic system, we used D_2_ as the hydrogen source and monitored the reaction using ^2^H NMR spectroscopy (**Figure** **3**a).^[^ ^37^ ^]^ The EtOH dispersion of HPP‐Pt NPs/C exhibited three characteristic peaks at ≈0.99 (methyl), 3.41 (methylene), and 4.88 (hydroxy) ppm, with no detectable free D_2_ signal. However, upon introduction of D_2_, we observed a significant enhancement of the peak at ≈4.88 ppm, indicating proton incorporation into the EtOH solvent to create O‐D species. This finding was confirmed by in situ ^2^H DRIFTS spectra (Figure 3b) with the appearance of an obvious O‐D band.^[^ ^38^ ^]^ These results indicate electron localization within the catalyst framework, comprising the Pt NPs and carbon support. As illustrated in Figure 3c, the proposed H_2_ activation mechanism involves: 1) Strong coordination of H_3_PO_2_ blocks the adsorption of unsaturated substrates while enabling H_2_ activation on Pt; 2) H_2_ activation generates electrons within the metal and carbon support, and protons are transferred to the EtOH solvent. Such a unique mechanism thus enables selective hydrogenation while preventing direct substrate‐catalyst interactions.

### 2.4 Noncontact Hydrogenation of Nitrobenzene via Electron and Proton Transfer

We conducted a detailed investigation of the elementary reactions in catalytic hydrogenation, focusing on electron and proton transfer processes.^[^ ^39^, ^40^ ^]^ Since no condensation products were detected during NB hydrogenation, our study concentrated exclusively on the direct hydrogenation pathway. The substrate hydrogenation pathway was examined stepwise in reverse order of PHA, NSB, and NB to elucidate the mechanism.

Initial studies of PHA conversion to AN revealed a critical challenge: the extremely slow hydrogenation rate of PHA (≈5.2%, 40 min, Figures S17 and S18, Supporting Information). This slow kinetics leads to significant accumulation of hydroxylamines, a well‐documented obstacle in the hydrogenation production of high‐purity amines.^[^ ^14^, ^41^, ^42^, ^43^ ^]^ To address this limitation, we employed vanadium‐mediated disproportionation, as vanadium species are known to effectively reduce the hydroxylamines accumulation.^[^ ^44^ ^]^ The introduction of VO(acac)_2_ into the EtOH solution of PHA under N_2_ atmosphere rapidly produced AXB and AN within ≈40 min, with a reaction rate ≈19 times faster than direct hydrogenation (Figures S17–S19, Supporting Information).^[^ ^10^ ^]^ Furthermore, when using HPP‐Pt NPs/C with vanadium promoters, PHA accumulation during NB hydrogenation became negligible (Figure 1f). These results demonstrate that the NB‐to‐AN conversion over HPP‐Pt NPs/C primarily occurs through NB hydrogenation to PHA, with minimal contribution from subsequent PHA hydrogenation to AN.

**Figure 4:**
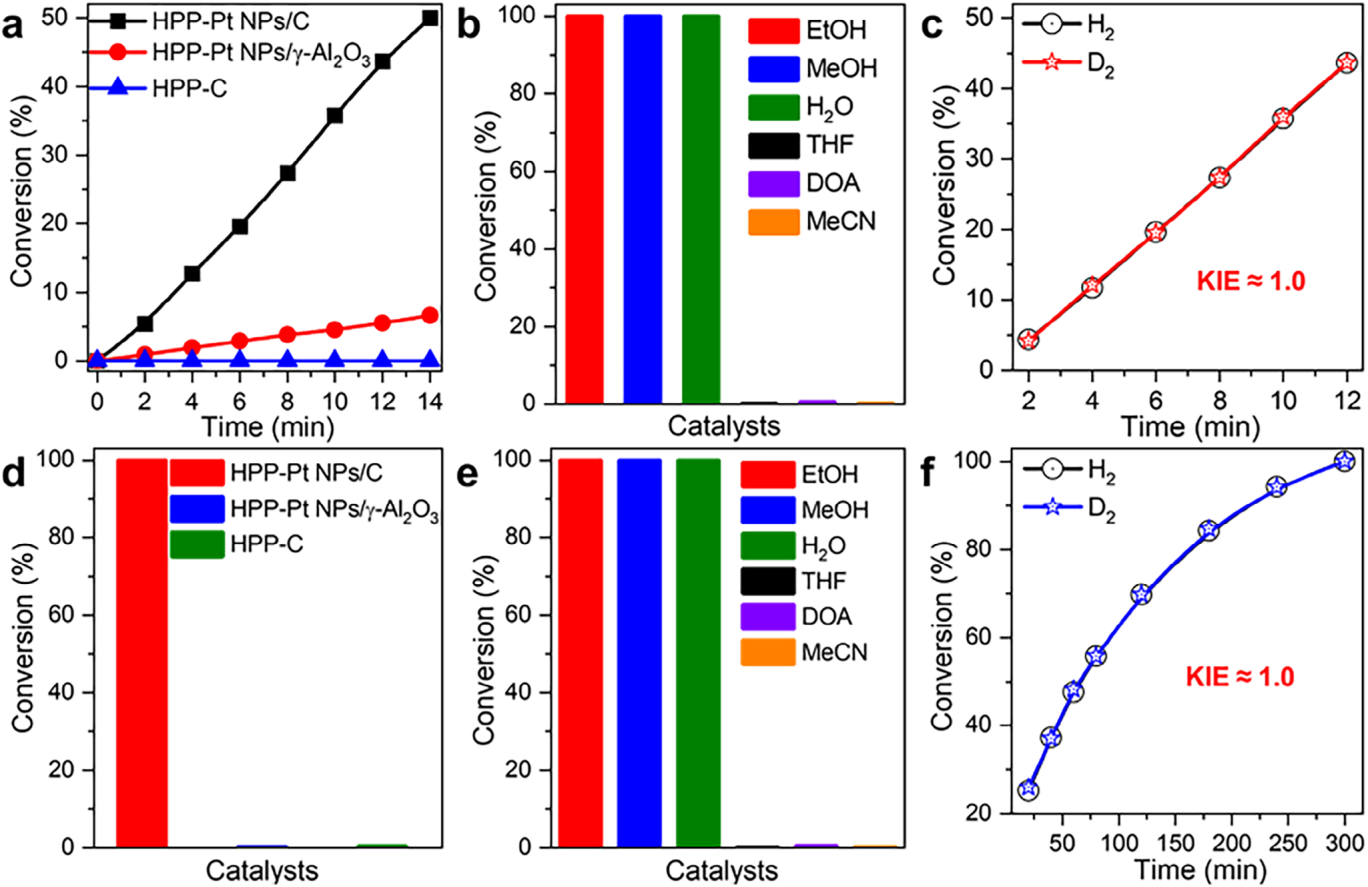
a) NSB hydrogenation catalyzed by HPP‐Pt NPs/C, HPP‐Pt NPs/γ‐Al_2_O_3_, and HPP‐C. b) NSB hydrogenation catalyzed by HPP‐Pt NPs/C in EtOH, MeOH, H_2_O, THF, DOA, and MeCN. c) KIE of NSB hydrogenation. d) NB hydrogenation catalyzed by HPP‐Pt NPs/C, HPP‐Pt NPs/γ‐Al_2_O_3_, and HPP‐C. e) NB hydrogenation catalyzed by HPP‐Pt NPs/C in EtOH, MeOH, H_2_O, THF, DOA, and MeCN. f) KIE of NB hydrogenation.

It is important to note that even with vanadium promoters, the disproportionation pathway for NB hydrogenation to AN still requires the conversion of NSB to PHA as a key intermediate step.^[^ ^44^ ^]^ We therefore investigated NSB hydrogenation to PHA. Using HPP‐Pt NPs/C (0.067 µmol Pt) as the catalyst, NSB was rapidly hydrogenated to AXB with high selectivity within ≈14 min (**Figure** **4**a; Figure S20, Supporting Information). This transformation involves two pathways: 1) direct hydrogenation of NSB to PHA, and 2) condensation between NSB and PHA to yield AXB (Figure S21, Supporting Information). Control experiments highlighted the critical role of conductive supports in the catalytic system. When γ‐Al_2_O_3_ (an electronically insulating material) was used as support, the H_3_PO_2_‐modified Pt NPs/γ‐Al_2_O_3_ catalyst (denoted HPP‐Pt NPs/γ‐Al_2_O_3_) exhibited an inferior activity, showing a sixfold reduction in NSB conversion rate compared to the carbon‐supported system (Figure 4a; Figures S22 and S23, Supporting Information). Meanwhile, the H_3_PO_2_‐modified XC‐72 support (denoted as HPP‐C) displayed negligible catalytic activity (Figure 4a; Figure S24, Supporting Information). These results demonstrate that the conductive carbon supports are essential for efficient electron transfer.

The residual activity observed with HPP‐Pt NPs/γ‐Al_2_O_3_ likely stems from electron transfer through ligand‐metal and support‐metal interfaces in this noncontact system (Figure 2b, blue line).^[^ ^45^, ^46^, ^47^ ^]^ Quantitative analysis reveals that electron transfer through carbon surfaces occurs ≈6 times faster than through ligand/support‐metal interfaces. To further compare electron transfer rates between different interfaces, we first synthesized monodisperse Pt nanocrystals (Pt NCs) through a hydrothermal reduction method in the presence of CO.^[^ ^48^ ^]^ Following ligand exchange, the amine ligands were replaced by H_3_PO_2_ (Figure S25, Supporting Information),^[^ ^49^ ^]^ yielding H_3_PO_2_‐modified Pt NCs (HPP‐Pt NCs) with an average particle size of ≈3.9 nm (Figure S26, Supporting Information). This HPP‐Pt NCs catalyst contains exclusively ligand‐metal interfaces. Subsequently, we prepared a comparative catalyst by supporting monodisperse Pt NCs on γ‐Al_2_O_3_ and removing the amine ligands by thermal treatment.^[^ ^49^ ^]^ After H_3_PO_2_ modification, the resulting HPP‐Pt NCs/γ‐Al_2_O_3_ catalyst showed a negligible change in Pt NCs particle size (Figures S27 and S28, Supporting Information) but now contained both ligand‐metal interfaces and support‐metal interfaces. In the hydrogenation of NSB, the HPP‐Pt NCs/γ‐Al_2_O_3_ catalyst exhibited ≈1.79‐fold faster activity than HPP‐Pt NCs under identical reaction conditions (Figure S29, Supporting Information). These results confirm that electron transfer in HPP‐Pt NCs/γ‐Al_2_O_3_ occurs through both ligand‐metal and support‐metal interfaces, with the former being ≈1.29 times more efficient than the latter.

Moreover, solvent studies provided insight into the proton transfer pathway. The HPP‐Pt NPs/C‐catalyzed NSB hydrogenation proceeded efficiently in protic solvents [e.g., ethanol (EtOH), methanol (MeOH), H_2_O] but showed negligible activity in non‐protic solvents [e.g., tetrahydrofuran (THF), 1,4‐dioxane (DOA), acetonitrile (MeCN)], demonstrating the essential role of protic solvents in facilitating proton transfer during the hydrogenation (Figure 4b; Figure S30, Supporting Information). Kinetic isotope effect (KIE) measurements yielded a value of ≈1.0 (Figure 4c; Figures S20 and S31, Supporting Information),^[^ ^10^, ^50^ ^]^ identifying electron transfer rather than proton transfer as RDS in this hydrogenation process.^[^ ^50^ ^]^

Since the rate of NSB hydrogenation to PHA is ≈67 times faster than that of NB to NSB (Figures 1f and 4a), the hydrogenation of NB to NSB is identified as RDS of the overall hydrogenation process. As shown in Figure 4d,e and Figures S32 and S33 (Supporting Information), NB can obtain electrons and protons from the carbon support and protic solvents, respectively. This is evidenced by the fact that neither HPP‐Pt NPs/γ‐Al_2_O_3_ nor HPP‐C can catalyze NB hydrogenation in EtOH, whereas HPP‐Pt NPs/C can effectively catalyze NB hydrogenation only in protic solvents, not in aprotic solvents. Furthermore, the rate‐determining nature of electron transfer is supported by the KIE of ≈1.0 observed in NB hydrogenation (Figure 4f; Figures S34 and S35, Supporting Information).

**Figure 5:**
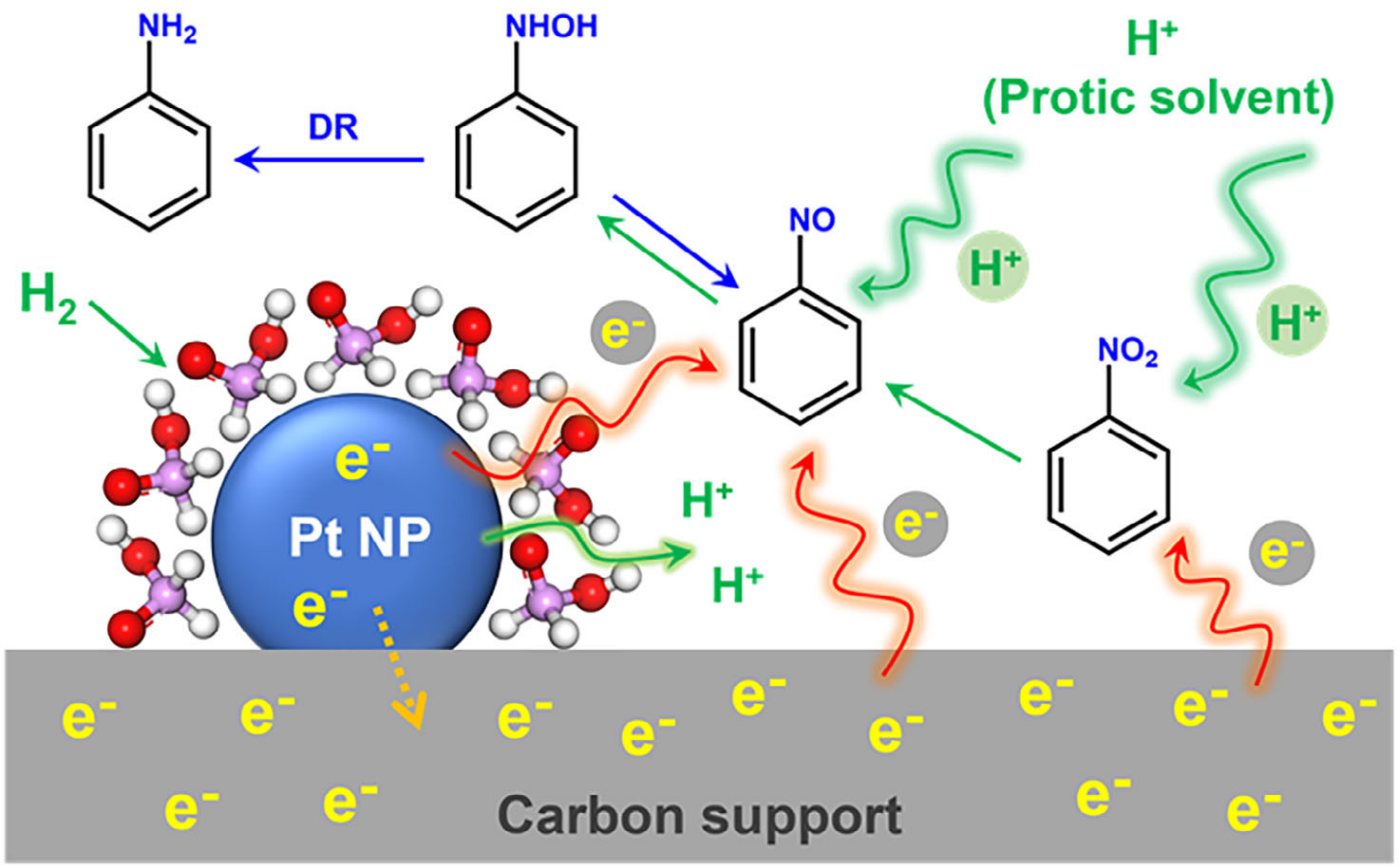
Illustration of the hydrogenation mechanism of NB to AN in noncontact catalytic hydrogenation. DR: disproportionation reaction (blue arrows).

The overall mechanism of NB hydrogenation to AN in the noncontact catalytic system is illustrated in **Figure** **5**. Given that NB hydrogenation to NSB is much slower than the subsequent hydrogenation of NSB to PHA, and that the disproportionation of PHA to AN is a more favorable pathway than PHA hydrogenation, the initial hydrogenation of NB to NSB constitutes the RDS of the entire reduction sequence. Proton transfer occurs in protic solvents during both NB‐to‐NSB and NSB‐to‐PHA transformations. Simultaneously, electron transfer, taking place at both the conducting carbon surfaces and ligand/support‐metal interfaces, plays a central role in driving the reaction. Notably, NB accepts electrons solely from the conducting carbon surfaces, whereas NSB can acquire electrons through both pathways, with electron transfer via carbon surfaces being the more effective route.

### 2.5 Exploring Potential of Noncontact Catalytic Hydrogenation

With the mechanistic insights, the developed noncontact catalytic system demonstrates broad applicability across various supported Pt or Pd NPs on carbon supports such as XC‐72, carbon nanotube (CNT), and graphene (GR), modified with ligands including hypophosphite and phosphite acid. Catalysts such as H_3_PO_2_‐modified Pd NPs/XC‐72, Pt NPs/CNT, and Pt NPs/GR exhibit catalytic behaviors similar to that of HPP‐Pt NPs/C, selectively hydrogenating NB to AN, while showing no activity for styrene hydrogenation under the same conditions (Figures S36 and S37, Supporting Information). Similarly, H_3_PO_3_‐modified Pt NPs/C displays analogous catalytic properties to HPP‐Pt NPs/C (Figures S36 and S37, Supporting Information).

Furthermore, H_3_PO_2_ was utilized to modify a commercial Pt NPs/C (2 wt.%) catalyst (Figure S38, Supporting Information). The application scope of the as‐obtained HPP‐Pt NPs/C‐com catalyst in the selective hydrogenation of nitroaromatics was investigated. As shown in **Table** **1**, HPP‐Pt NPs/C‐com exhibited significantly enhanced catalytic performance in the selective hydrogenation of substituted nitroaromatics to functional aromatic amines (Table 1, Entries 1–16). It is worthy of note that, even in the presence of halogen atoms (F, Cl, Br, and I) or unsaturated functional groups (C═C, C═O, C═N, and C≡N) in the substituent groups, the nitroaromatics were efficiently converted into their corresponding amines with negligible side reactions.

**Table 1:** The hydrogenation of nitroaromatics and quinones over HPP‐Pt NPs/C‐com catalyst.

Entry	Substrate	Product	Yield [%]
1	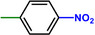	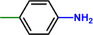	>99.9
2	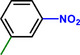	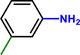	>99.9
3	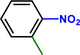	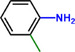	>99.9
4	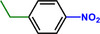	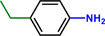	>99.9
5	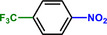	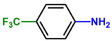	>99.9
6	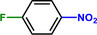	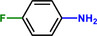	>99.9
7	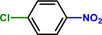	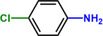	>99.9
8	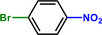	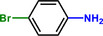	>99.9
9	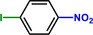	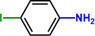	>99.5
10	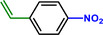	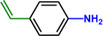	>99.0
11	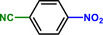	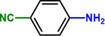	>99.9
12	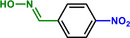	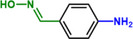	>99.0
13	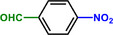	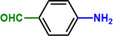	>99.9
14	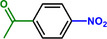	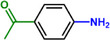	>99.9
15	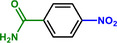	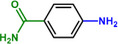	>99.9
16	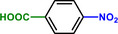	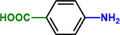	>99.9
17	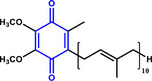	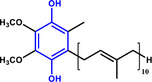	>99.9
18	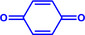	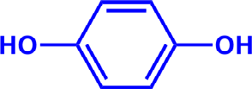	>99.9
19	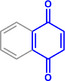	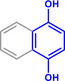	>99.9
20	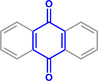	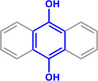	>99.9
21	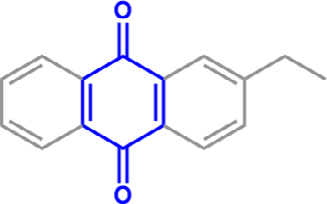	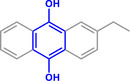	>99.9

Reaction conditions: HPP‐Pt NPs/C‐com (2 µmol Pt), substrates (1.0 mmol), 0.3 MPa H_2_, 60 °C, 10 mL EtOH, 6 h.

Importantly, the noncontact catalytic approach developed in this study is conceptually analogous to biological hydrogenation systems, where spatially separated electron and proton transfer pathways enable precise hydrogenation of substrates. This mechanistic parallel suggests broad potential for selectively hydrogenating biologically relevant oxidants, including quinones and their structural analogues. As demonstrated in our catalytic evaluations (Table 1, Entries 17–21), the HPP‐Pt NPs/C‐com system achieves exceptional selectivity (>95%) in the hydrogenation of coenzyme Q10, a challenging transformation due to the molecule's redox‐sensitive ubiquinone moiety and extended conjugated system. The bioinspired strategy extends to structurally diverse quinones (Entries 18–21), underscoring its generality for medicinally relevant hydrogenations where conventional catalysts often fail. The system's performance with coenzyme Q10 and its analogue quinones, biomolecules much larger than nitrobenzene, particularly highlights its advantage over contact‐dependent mechanisms.

## 3 Conclusion

A noncontact catalytic system has been developed by the surface modification of Pt or Pd nanoparticles supported on conductive supports with (hypo)phosphorous acid. The resulting isolation layer prevents direct contact between unsaturated substrates and the active metal surface while still permitting H_2_ activation. In this system, H_2_ activation generates electrons and protons on the carbon‐supported Pt/Pd catalysts and in the protic solvents, respectively. The hydrogenation of nitroaromatics to hydroxylamines proceeds via electron transfer, where the nitro compounds acquire electrons from the carbon support surface, while the nitroso intermediate can also receive electrons from the ligand/support‐metal interface. Protons transfer with the help of protic solvents. However, further electron transfer from the catalyst surface to hydroxylamine is unfavorable, making disproportionation a more efficient pathway. By elucidating the catalytic mechanism, the heterogeneous catalytic system was successfully expanded to various metals, carbon supports, and ligands. The H_3_PO_2_‐modified commercial Pt/C catalyst demonstrated high efficiency in the selective hydrogenation of nitroaromatics to amines. Moreover, the catalytic system exhibits functional similarities to biocatalytic hydrogenation processes, enabling the selective hydrogenation of biological oxidants, such as coenzyme Q10 and its analogue quinones.

## Conflict of Interest

The authors declare no conflict of interest.

## Supporting information



Supporting Information

## Data Availability

The data that support the findings of this study are available in the Supporting Information of this article.
