# Supplementary material for: Decoupling Electron and Proton Transfer in Noncontact Catalytic Hydrogenation of Nitroaromatics
Source: Adv Sci (Weinh). 2025 Aug 28;12(43):e11391. doi: 10.1002/advs.202511391 (PMC12631828; doi:10.1002/advs.202511391)
Supplement: Supplementary file 1 — Supporting Information [file ADVS-12-e11391-s001.pdf]

Supporting Information

Decoupling Electron and Proton Transfer in Noncontact Catalytic Hydrogenation of Nitroaromatics

Zhongyin Liang, Qingyuan Wu, Zhe Yang, Nanfeng Zheng**

Z. Y. Liang, Q. Y. Wu, Z. Yang, N. F. Zheng
New Cornerstone Science Laboratory, State Key Laboratory for Physical Chemistry of Solid Surfaces, Collaborative Innovation Center of Chemistry for Energy Materials, and National & Local Joint Engineering Research Center for Preparation Technology of Nanomaterials, College of Chemistry and Chemical Engineering, Xiamen University, Xiamen 361005, China
E-mail: nfzheng@xmu.edu.cn; qywu@xmu.edu.cn

N. F. Zheng
Innovation Laboratory for Sciences and Technologies of Energy Materials of Fujian Province (IKKEM), Xiamen 361102, China

Z. Y. Liang and Q. Y. Wu contributed equally to this work

Experimental Details

Materials. Chloroplatinic acid (H_2PtCl_6, A.R.), platinum diacetylacetonate ($C_{10}H_{16}O_4Pt$, Pt(acac)$_2$, A.R.), hypophosphorous acid (H_3PO_2, A.R.), phosphorous acid (H_3PO_3, A.R.), carbon nanotubes (CNT, A.R.), graphene (GR, A.R.), aluminum oxide (γ-Al_2O_3, A.R.), cyclohexanone oxime ($C_6H_{11}NO$, A.R.), benzonitrile (C_7H_5N, A.R.), benzaldehyde (C_7H_6O, A.R.), benzoic acid ($C_7H_6O_2$, A.R.), benzyl alcohol (C_7H_8O, A.R.), styrene (C_8H_8, A.R.), phenylacetylene (C_8H_6, A.R.), acetophenone (C_8H_8O, A.R.), 4-octyne (C_8H_{14}, A.R.), 1-phenylpropyne (C_9H_8, A.R.), linalool ($C_{10}H_{18}O$, A.R.), N,1-diphenylmethanimine ($C_{13}H_{11}N$, A.R.) were purchased from Shanghai Aladdin Biochemical Technology Co., Ltd (Shanghai, China). Methanol (CH_4O, A. R.), ethanol (C_2H_5OH, A.R.), N,N-dimethylformamide (C_3H_7NO, A.R.), oleylamine ($C_{18}H_{37}N$, A.R.) and butylamine ($C_4H_{11}N$, A.R.) were purchased from Sinopharm Chemical Reagent Co. Ltd. (Shanghai, China). Methanol-D4 (CD_4O, A.R.), acetonitrile (CH_3CN, A.R.), tetrahydrofuran (C_4H_8O, A.R.), 1,4-dioxane ($C_4H_8O_2$, A.R.), nitrobenzene ($C_6H_5NO_2$, A.R.), nitrosobenzene (C_6H_5NO, A.R.), phenylhydroxylamine (C_6H_7NO, A.R.), 4-nitroaniline ($C_6H_6N_2O_2$, A.R.), 4-fluornitrobenzol ($C_6H_4FNO_2$, A.R.), 4-chloronitrobenzene ($C_6H_4ClNO_2$, A.R.), 4-nitrotoluene ($C_7H_7NO_2$, A.R.), 3-nitrotoluene ($C_7H_7NO_2$, A.R.), 2-nitrotoluene ($C_7H_7NO_2$, A.R.), 4-nitrobenzotrifluoride ($C_7H_4F_3NO_2$, A.R.), 4-nitrobenzaldoxime ($C_7H_6N_2O_3$, A.R.), 4-ethylnitrobenzene ($C_8H_9NO_2$, A.R.), 4-ethynylnitrobenzene ($C_8H_5NO_2$, A.R.), 4-nitrostyrene ($C_8H_7NO_2$, A.R.), 4-bromonitrobenzene ($C_6H_4BrNO_2$, A.R.), 4-iodonitrobenzene ($C_6H_4INO_2$, A.R.), 4-nitroacetophenone ($C_8H_7NO_3$, A.R.), 4-nitrobenzonitrile ($C_7H_4N_2O_2$, A.R.), 4-nitrobenzoic acid ($C_7H_5NO_4$, A.R.), 4-nitrobenzamide ($C_7H_6N_3O_3$, A.R.), 4-nitrobenzaldehyde ($C_7H_5NO_3$, A.R.), 1,4-benzoquinone ($C_6H_4O_2$, A.R.), 1,4-naphthoquinone ($C_{10}H_6O_2$, A.R.), anthraquinone ($C_{14}H_8O_2$, A.R.) and 2-ethylanthraquinone ($C_{16}H_{12}O_2$, A.R.) were purchased from Energy Chemical (Shanghai, China). H_2 (99.999%), N_2 (99.999%), D_2 (99.999%), Ar (99.999%), CO (99.999%), NO (5 vol%)/Ar and CO (5 vol%)/Ar were purchased from Linde Gas. The water used in all experiments was ultrapure (18.25 MΩ·cm). XC-72 was purchased from Cabot Corporation (Boston, USA). All reagents were used without further purification.

Synthesis of $[Pt_3(CO)_3(\mu_2\text{-}CO)_3]_5^{2-}$ clusters. The clusters were prepared following a modified method reported by Zheng et al.[1] In a typical synthesis, the aqueous solution of H_2PtCl_6 (0.1 M, 0.5 mL) mixed with dimethylformamide (DMF, 10 mL) was reduced at 50°C for 12 h in a glass pressure vessel which was charged with 0.1 MPa CO. The obtained blue-green solution was used for further preparation of supported Pt nanoparticles without any other treatments.

Synthesis of Pt NPs/C. For the preparation of Pt NPs/C, 1 mL of the above-mentioned solution was firstly diluted with 4 mL of DMF. The diluted solution was then mixed with 50 mg XC-72 under vigorous stirring. The mixture was heated at 30°C and kept at this temperature for a period of 12 h. The product was collected by centrifugation, washed three times with ethanol, and dried in a vacuum oven at 60°C for further use.

Synthesis of Pt NPs/γ-Al_2O_3. The synthesis of Pt NPs/γ-Al_2O_3 followed the same protocol as the synthesis of Pt NPs/C, with the substitution of the XC-72 with γ-Al_2O_3.

Synthesis of H_3PO_2 modified Pt NPs/C. In a typical modification, the Pt NPs/C (20 mg) was ultrasonically redispersed in 2 mL of ethanol in a 48 mL glass pressure vessel. Subsequently, H_3PO_2 aqueous solution (1 mL, 0.21 mmol/mL) was added dropwise and the mixture was stirred at room temperature for 30 min. The above solution was collected for further use.

Synthesis of H_3PO_2 modified Pt NPs/γ-Al_2O_3. The modification of Pt NPs/γ-Al_2O_3 followed the identical protocol, with the substitution of the Pt NPs/C with Pt NPs/γ-Al_2O_3.

Synthesis of H_3PO_2 modified XC-72. The modification of XC-72 followed the identical protocol, with the substitution of the Pt NPs/C with XC-72.

Synthesis of Pt NCs. The Pt(acac)$_2$ (40 mg, 0.1 mmol Pt) was added to a glass pressure vessel containing 20 mL of oleylamine. The vessel was then placed in a 70°C water bath and stirred until a clear and transparent yellow solution was formed. The vessel was purged with CO and the CO pressure was maintained at 1 bar. Subsequently, the vessel was transferred to an oil bath that had been preheated to 180°C. Thereafter, the contents of the vessel were stirred vigorously for 40 min. Following a period of natural cooling to room temperature, an appropriate amount of ethanol was added and the mixture was subjected to centrifugation in order to collect the Pt NCs.

Synthesis of H_3PO_2 modified Pt NCs (HPP-Pt NCs). After precipitation of the prepared Pt nanocrystals using excess ethanol, the solids were re-dispersed in 40 mL of n-butylamine at a concentration of approximately 0.5 mg Pt/mL, forming a uniformly dispersed Pt nanoparticle sol. The dispersion was stirred at room temperature for 3 days. Ethanol was then added to induce precipitation, and the solids were collected by centrifugation. The precipitate was washed several times with ethanol to remove excess n-butylamine. The Pt nanocrystals were subsequently re-dispersed in 10 mL of an aqueous H_3PO_2 solution at a concentration of approximately 2 mg Pt/mL. The mixture was stirred at room temperature for 3 days to ensure complete ligand exchange, followed by centrifugation and at least three ethanol washes. The resulting H_3PO_2-modified Pt nanocrystals were dried under vacuum overnight.

Synthesis of Pt NCs/γ-Al_2O_3. After precipitation of the prepared Pt nanocrystals using excess ethanol, the solids were re-dispersed in 40 mL of n-butylamine at a concentration of approximately 0.5 mg Pt/mL, forming a uniformly dispersed Pt nanoparticle sol. Subsequently, approximately 1 g of γ-Al_2O_3 was added to the above dispersion, and the mixture was stirred at room temperature for 3 days. The solid product was collected by centrifugation, followed by multiple ethanol washes to remove excess n-butylamine. The resulting solid was vacuum-dried overnight. After being ground into a fine powder, the sample was slowly heated in air (50 mL/min) to 200 °C over 6 hours, held at 200 °C for 1 hour, and then cooled to room temperature.

Synthesis of H_3PO_2 modified Pt NCs/γ-Al_2O_3 (HPP-Pt NCs/γ-Al_2O_3). In a typical procedure, 20 mg of Pt NCs/γ-Al_2O_3 was re-dispersed in a glass pressure vessel using 2 mL of ethanol and ultrasonication, followed by dilution with 48 mL of deionized water. Subsequently, H_3PO_2 aqueous solution (1 mL, 0.21 mmol/mL) was added dropwise and the mixture was stirred at room temperature for 30 min. The resulting suspension was collected for further use.

Synthesis of Pd NPs/C, Pt NPs/CNT and Pt NPs/GR. For the synthesis of Pd NPs/C (2 wt%), 500 mg of XC-72 was ultrasonically dispersed into 60 mL of water in a 100 mL serum bottle. After stirring for 4 h, 4 mL of H_2PdCl_4 aqueous solution (3.2 mg·mL^{-1}) was added. Then, stirring was continued for 4 h, followed by the addition of 4 mL of freshly prepared $NaBH_4$ aqueous solution (1 mg·mL^{-1}). After another 4 h of stirring, the product was separated by centrifugation and further purified with water 3 times. Finally, the precipitate was dried overnight at 60ºC. For

the synthesis of Pt NPs/CNT (2 wt%), the synthesis process followed the same protocol as the synthesis of Pd NPs/C, with the substitution of the XC-72 and H_2PdCl_4 aqueous solution with CNT and H_2PtCl_6 aqueous solution (5 mL, 4.25 mg·mL^{-1}), respectively. For synthesis of Pt NPs/GR (2 wt%), the synthesis process followed the same protocol as the synthesis of Pt NPs/CNT, with the substitution of the CNT with GR.

Synthesis of H_3PO_2 modified Pd NPs/C, Pt NPs/CNT, Pt NPs/GR. The modification followed the identical protocol, with the substitution of the Pt NPs/C with Pd NPs/C, Pt NPs/CNT or Pt NPs/GR.

Synthesis of H_3PO_3 modified Pt NPs/C. The modification followed the identical protocol, with the substitution of H_3PO_2 with H_3PO_3.

Synthesis of H_3PO_2 modified commercial Pt NPs/C. The modification of commercial Pt NPs/C (2 wt%) followed the identical protocol, with the substitution of the Pt NPs/C with commercial Pt NPs/C.

STEM and TEM characterizations. The analysis of STEM and TEM was conducted using a JEOL JEM-F200 transmission electron microscope operated at 200 kV. The samples were prepared by depositing an ethanol dispersion onto 300-mesh carbon-coated copper grids, followed by immediate solvent evaporation.

Nuclear magnetic resonance (NMR) Characterizations. ^{1}H NMR and ^{2}H NMR spectra were recorded at room temperature on an AVANCE III 500 MHz spectrometer, with TMS and the solvent residual signal used as internal references. Oxygen-isolated ^{1}H NMR samples were prepared following the method reported by Zheng et al.[2] All NMR data were processed using MestReNova software.

FTIR characterizations. In situ FTIR spectroscopy was performed using a Thermo Fisher IS50 spectrometer equipped with an MCT detector. All adsorption experiments were conducted in a Harrick in-situ chamber. For NB adsorption studies, following the loading of the HPP-Pt NPs/C, the chamber was purged with Ar (30°C, 10 min). NB vapor (carried by Ar) was then introduced for 10 min, followed by Ar purging prior to measurement. For CO adsorption studies, the HPP-Pt NPs/C was pretreated with pure Ar prior to exposure to a CO/Ar mixture for 10 min.

Spectra were collected post-Ar flushing. For NO adsorption studies, the HPP-Pt NCs was pretreated with pure Ar prior to exposure to a NO/Ar mixture for 10 min. Spectra were collected post-Ar flushing. The same procedure was applied to the HPP-Pt NCs/γ-Al_2O_3. For O-D detection, the FTIR spectra were collected by a thin-liquid film method using CaF_2 windows, in which the samples were deposited as thin liquid films on CaF_2 windows.

Powder X-ray diffraction (XRD) characterizations. XRD experiments were conducted on Rigaku Ultima IV using Cu Kα radiation. The operation voltage and current were 40 kV and 30 mA, respectively. The scanning speed was set as 10 °/min.

Catalytic evaluations

NB hydrogenation

NB hydrogenation catalyzed by Pt NPs/C: All catalytic reactions were conducted using a 48 mL glass pressure vessel equipped with a magnetic stirrer to eliminate the influence of mass transfer. In a typical hydrogenation experiment, the Pt NPs/C catalyst (2 μmol Pt) was first ultrasonically dispersed in 2 mL of ethanol and mixed with 1 mmol of NB. Subsequently, 7 mL of ethanol was added to the mixture, followed by the addition of 1 mL of $VO(acac)_2$ ethanol solution (2.65 mg). The system was then purged with N_2 for 2 min to completely remove any residual oxygen from the reactor. The reaction was then carried out under 3 bar of H_2 while maintaining a constant temperature of 60°C. Throughout the reaction progress, 200 μL aliquots were periodically withdrawn for analysis by ^1H NMR spectroscopy.

NB hydrogenation catalyzed by HPP-Pt NPs/C, HPP-Pt NPs/γ-Al_2O_3 or HPP-C: The catalytic process is analogous to that of NB hydrogenation catalyzed by Pt NPs/C, with the exception that HPP-Pt NPs/C, HPP-Pt NPs/γ-Al_2O_3 or HPP-C is utilized in place of Pt NPs/C.

NB hydrogenation in different solvent: The catalytic process is analogous to that of NB hydrogenation catalyzed by HPP-Pt NPs/C, with the exception that methanol, water, tetrahydrofuran, acetonitrile or 1,4-dioxane is utilized in place of ethanol.

KIE test: The catalytic process is analogous to that of NB hydrogenation catalyzed by HPP-Pt NPs/C, with the exception that D_2 is utilized in place of H_2.

Catalyst recyclability: To evaluate catalyst recyclability, after the first cycle was carried out for 120 min, another 1 mmol portion of fresh NB was added to the reactor without isolating or treating the catalyst for further hydrogenation. We repeated this process for five cycles to assess the catalyst's stability and consistent performance.

Different substrates hydrogenation catalyzed by HPP-Pt NPs/C-com

The catalytic process is analogous to that of NB hydrogenation catalyzed by HPP-Pt NPs/C, except that different substrates and HPP-Pt NPs/C-com are utilized in place of NB and HPP-Pt NPs/C, respectively.

PHA hydrogenation and disproportionation

PHA hydrogenation catalyzed by HPP-Pt NPs/C: The catalytic process is analogous to that of NB hydrogenation catalyzed by HPP-Pt NPs/C, with PHA is utilized in place of NB.

PHA disproportionation catalyzed by VO(acac)$_2$ or HPP-Pt NPs/C: The VO(acac)$_2$ (2.65 mg) or HPP-Pt NPs/C (2 μmol Pt) was first ultrasonically dispersed in 1 mL of ethanol. Thereafter, 9 mL of ethanol was added to the mixture, followed by the addition of 1 mL of PHA ethanol solution (1 mmol). The system was then purged with N_2 for 2 min to completely remove any residual oxygen from the reactor. The reaction was carried out under 3 bar N_2 while maintaining a constant temperature of 60°C. Throughout the reaction progress, we periodically withdrew 200 μL aliquots for analysis by NMR spectroscopy to monitor conversion rates.

NSB hydrogenation

NSB hydrogenation catalyzed by HPP-Pt NPs/C: The catalytic process is analogous to that of NB hydrogenation catalyzed by HPP-Pt NPs/C, except that HPP-Pt NPs/C (0.067 μmol Pt) and NSB (1 mmol) are utilized.

NSB hydrogenation catalyzed by HPP-Pt NPs/γ-Al$_2$O$_3$ or HPP-C: The catalytic process is analogous to that of NSB hydrogenation catalyzed by HPP-Pt NPs/C, except that HPP-Pt NPs/γ-Al_2O_3 (0.067 μmol Pt) or HPP-C are utilized.

NSB hydrogenation catalyzed by HPP-Pt NCs/γ-Al$_2$O$_3$ or HPP-Pt NCs: The catalytic process is analogous to that of NSB hydrogenation catalyzed by HPP-Pt NPs/C, except that HPP-Pt NCs/γ-Al_2O_3 (0.067 μmol Pt) or HPP-Pt NCs are utilized.

NSB hydrogenation in different solvent: The catalytic process is analogous to that of NSB hydrogenation catalyzed by HPP-Pt NPs/C, with the exception that methanol, water, tetrahydrofuran, acetonitrile or 1,4-dioxane is utilized in place of ethanol.

KIE test: The catalytic process is analogous to that of NSB hydrogenation catalyzed by HPP-Pt NPs/C, with the exception that D_2 is utilized in place of H_2.

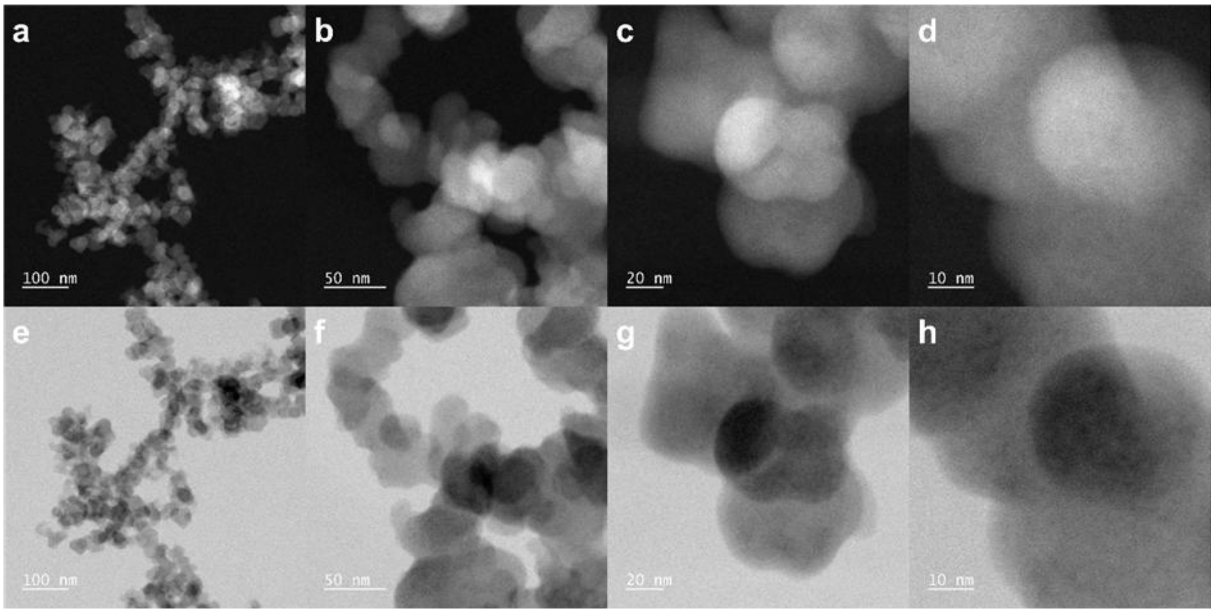

Figure S1. STEM (a-d) and TEM (e-h) images of XC-72.

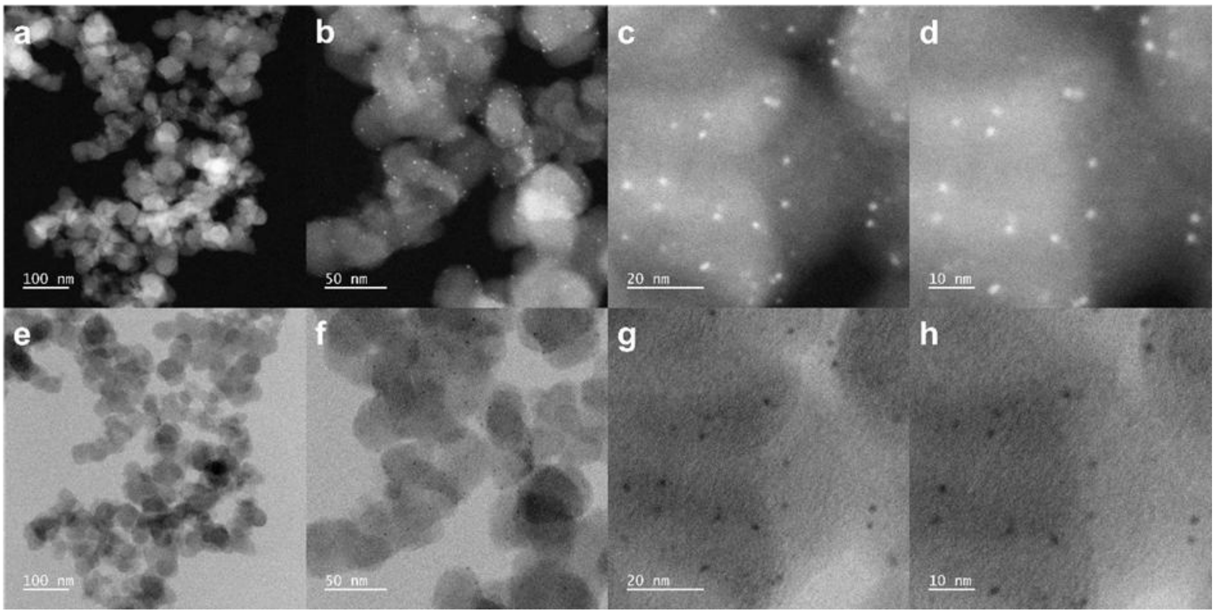

Figure S2. STEM (a-d) and TEM (e-h) images of Pt NPs/C. Small and uniform Pt NPs are supported on XC-72.

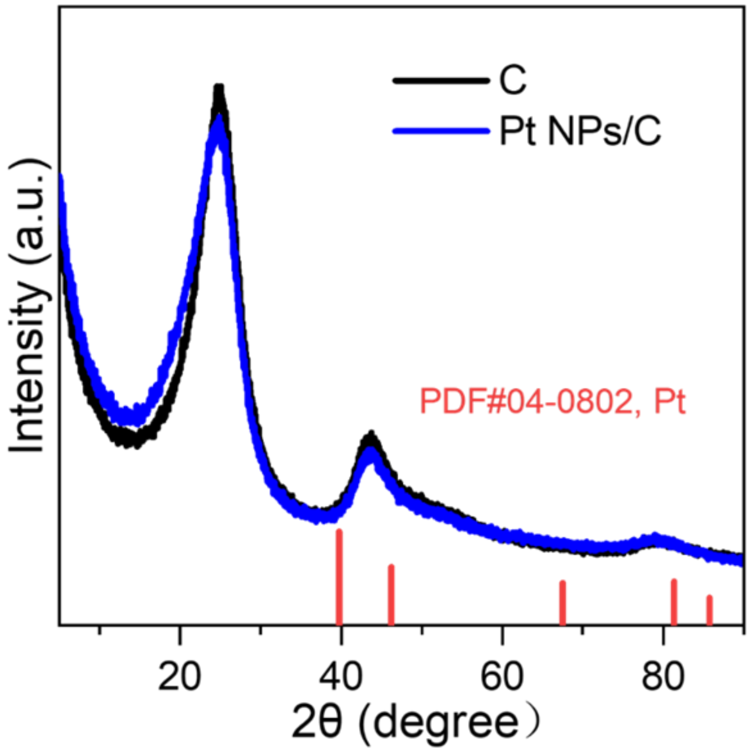

Figure S3. XRD patterns of XC-72 and Pt NPs/C. No large Pt NPs are found.

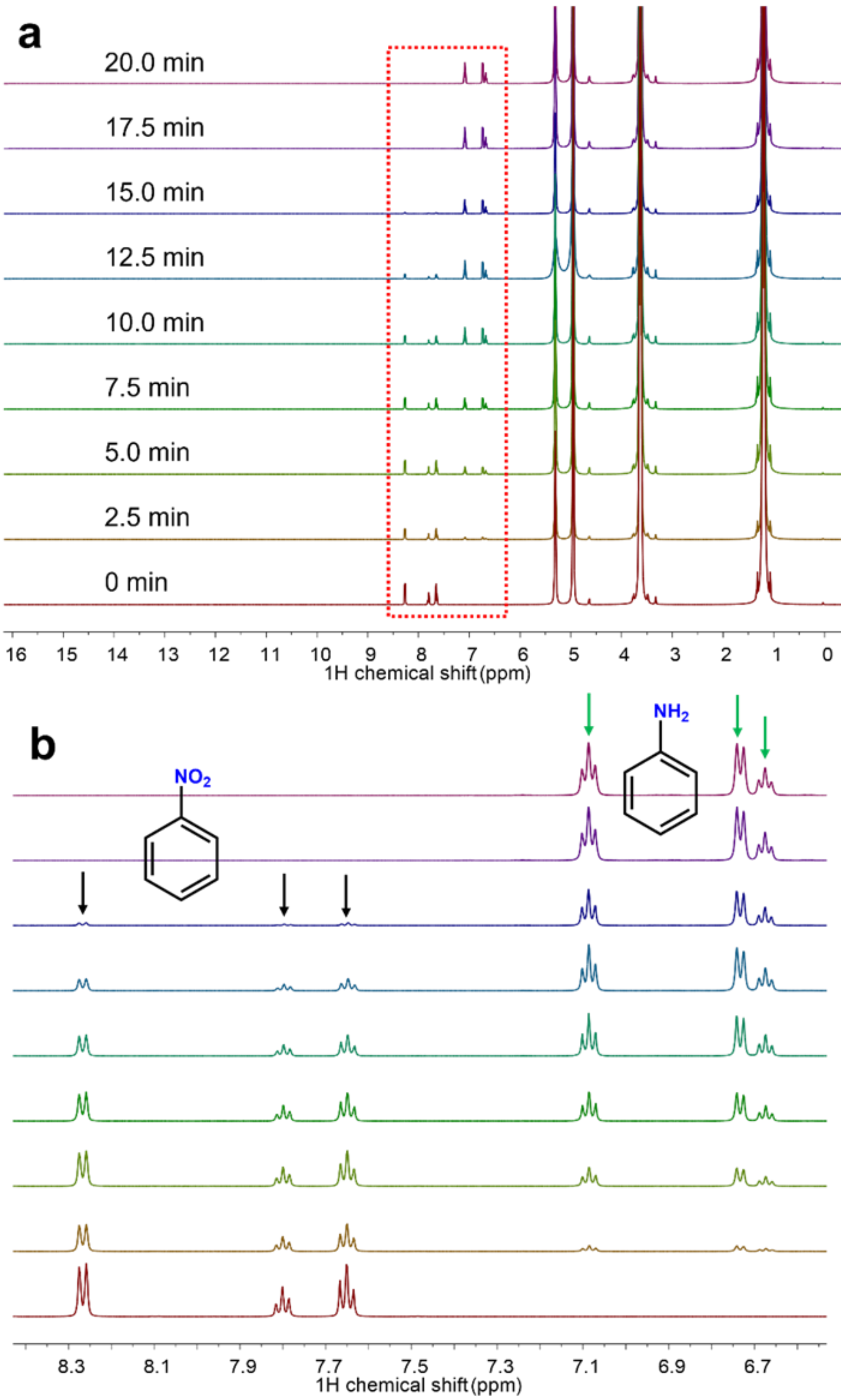

Figure S4. Time-dependent hydrogenation of NB over Pt NPs/C monitored by ^{1}H NMR spectroscopy. (a) Oxygen-isolated ^{1}H NMR spectra (in CD_3OD) of the reaction mixture in EtOH at different time points. (b) Magnified view of the aromatic region (boxed area in a), showing characteristic proton signals of NB (black arrows) and AN (green arrows).

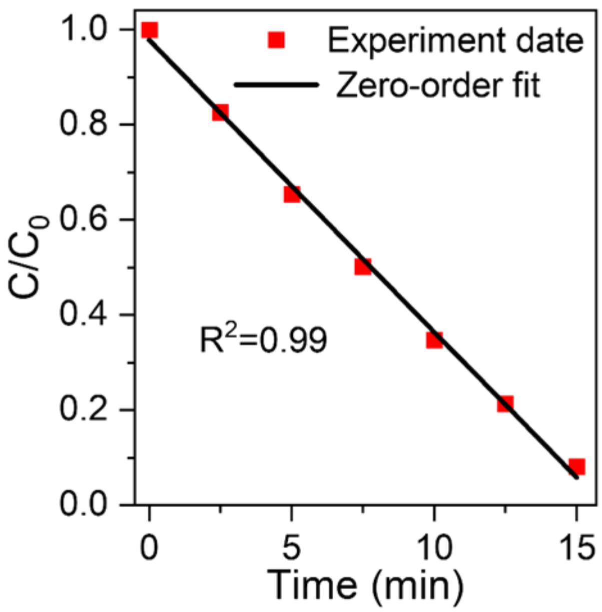

Figure S5. The zero-order kinetic relationship for NB hydrogenation to AN on Pt NPs/C.[3]

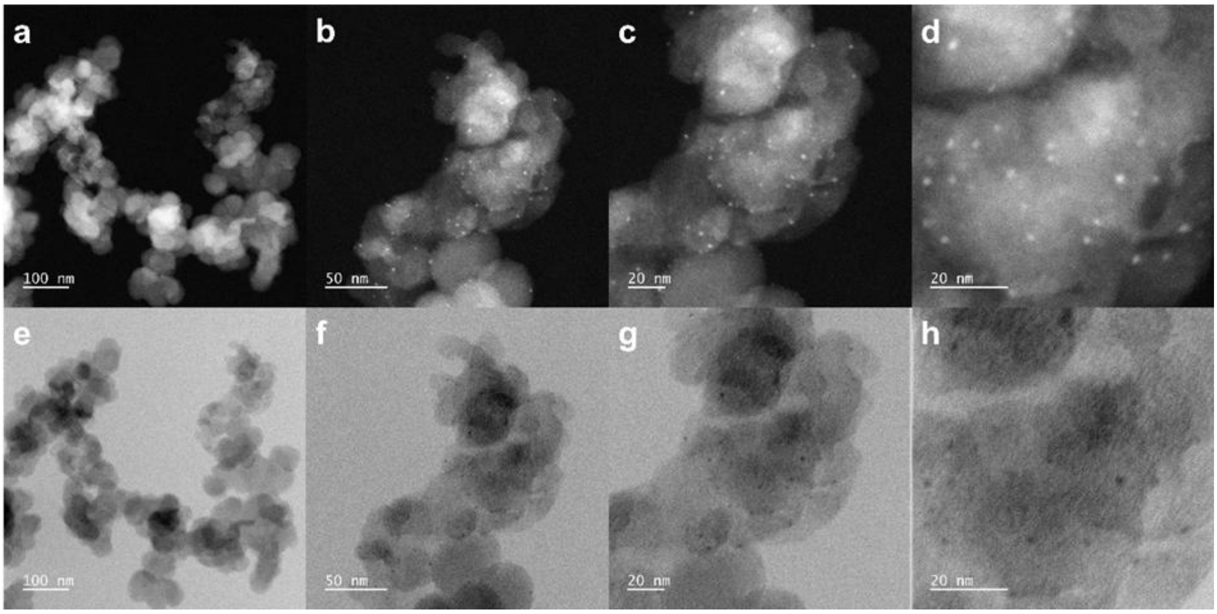

Figure S6. STEM (a-d) and TEM (e-h) images of HPP-Pt NPs/C. The modification of H_3PO_2 does not affect the particle size of Pt NPs.

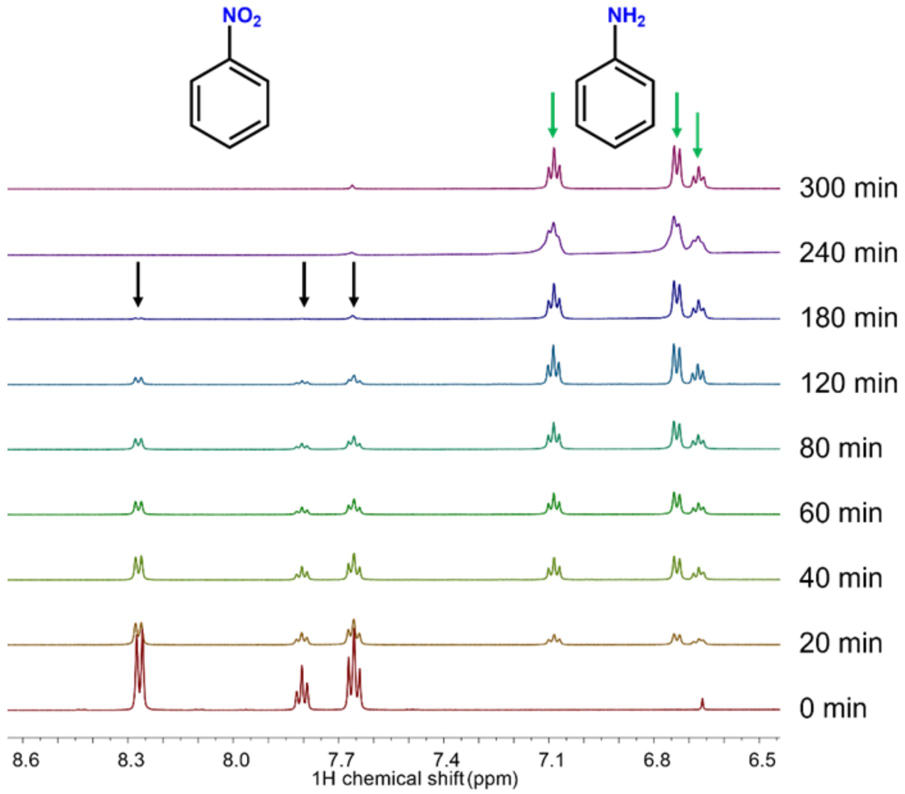

Figure S7. Time-dependent hydrogenation of NB (black arrows) to AN (green arrows) over HPP-Pt NPs/C monitored by ^{1}H NMR spectroscopy. Oxygen-isolated ^{1}H NMR spectra (in CD_3OD) of the reaction mixture in EtOH at different time points. Because the solvent NMR signal peaks are similar to those in Fig. S4a (0-6 ppm interval), this region is not shown for the sake of simplicity.

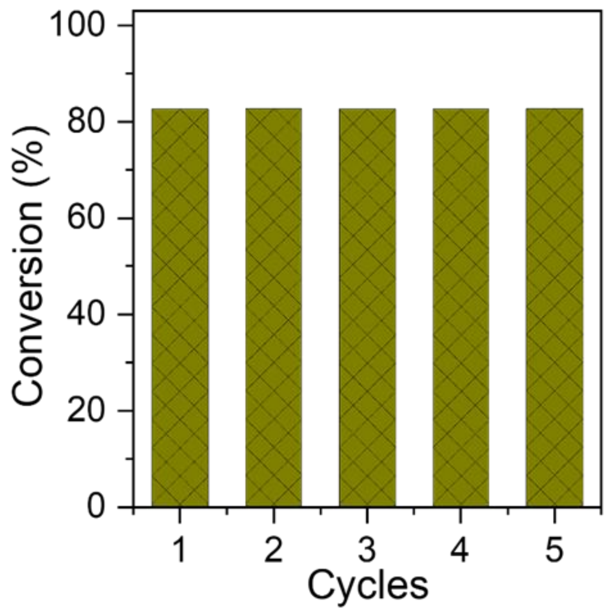

Figure S8. Stability test of HPP-Pt NPs/C for the hydrogenation of NB to AN. Reaction time: 120 min.

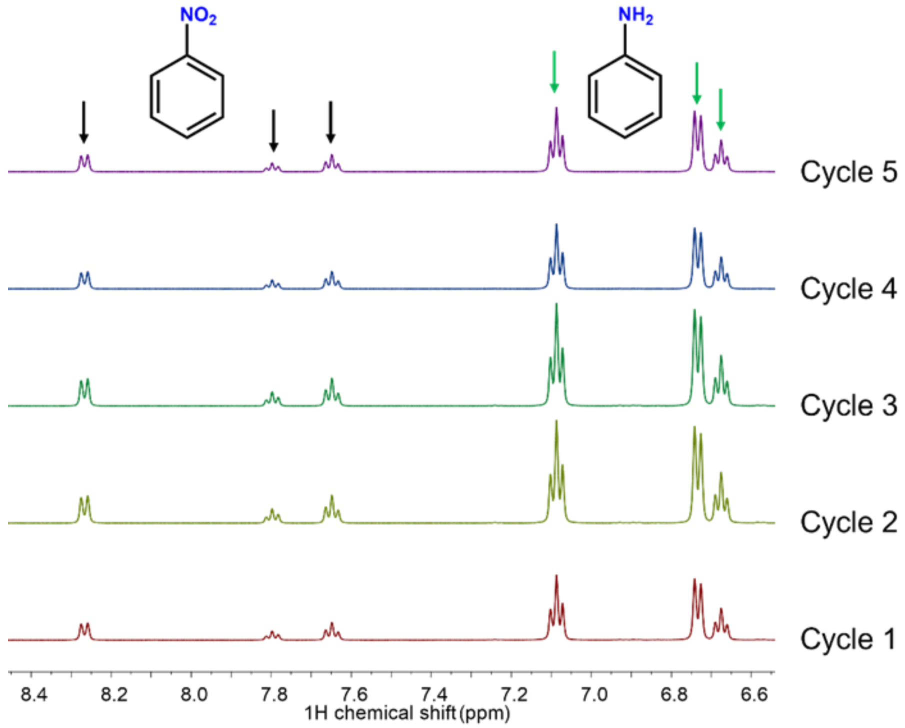

Figure S9. Oxygen-isolated ^{1}H NMR spectra (in CD_3OD) of the reaction mixture in EtOH at different time cycles. Black and green arrows indicate NB and AN, respectively.

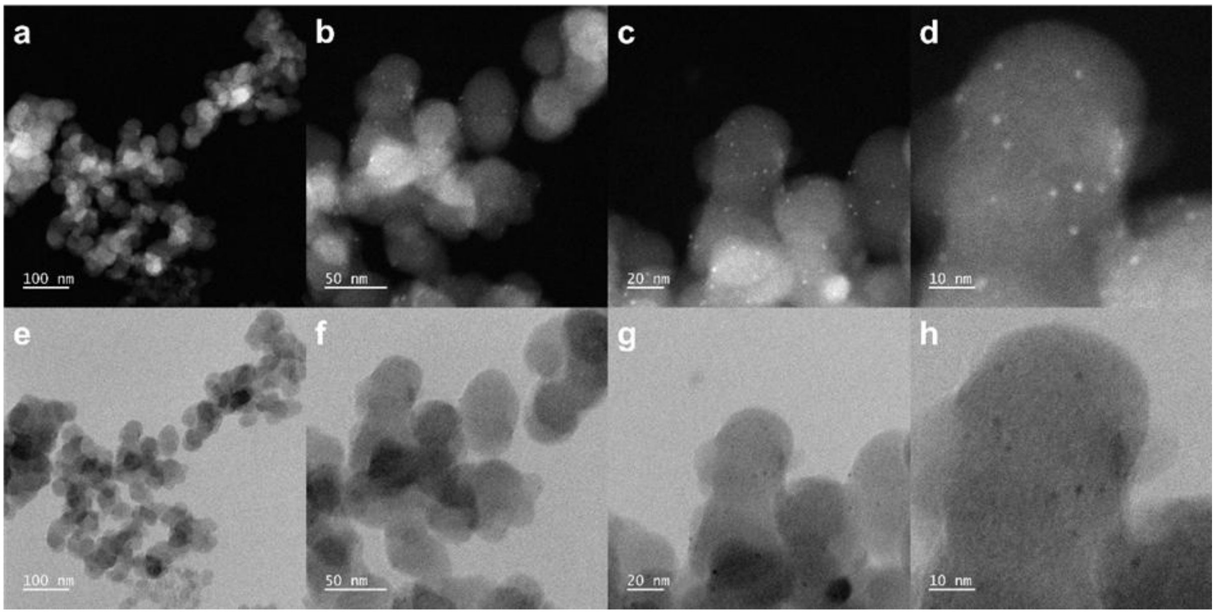

Figure S10. STEM (a-d) and TEM (e-h) images of HPP-Pt NPs/C after stability test. No observable size change of Pt NPs is found.

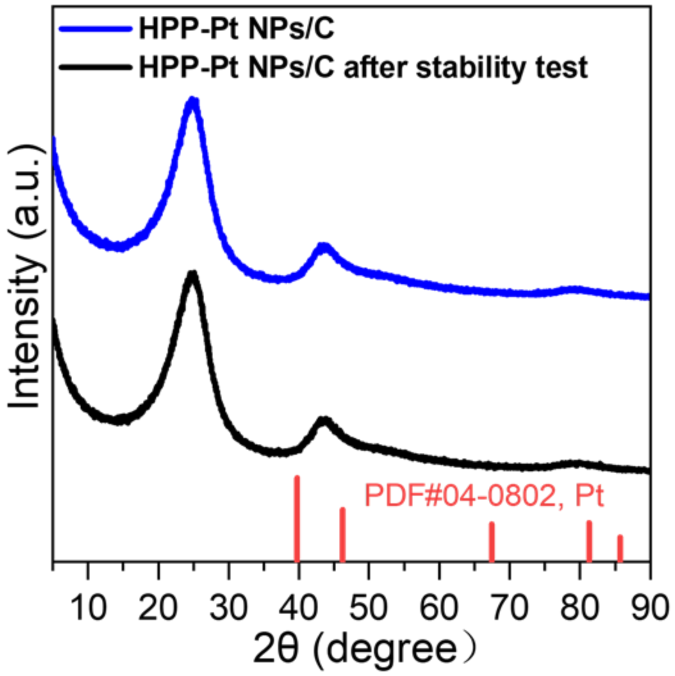

Figure S11. XRD patterns of HPP-Pt NPs/C and HPP-Pt NPs/C after stability test. No large Pt NPs are found after stability test.

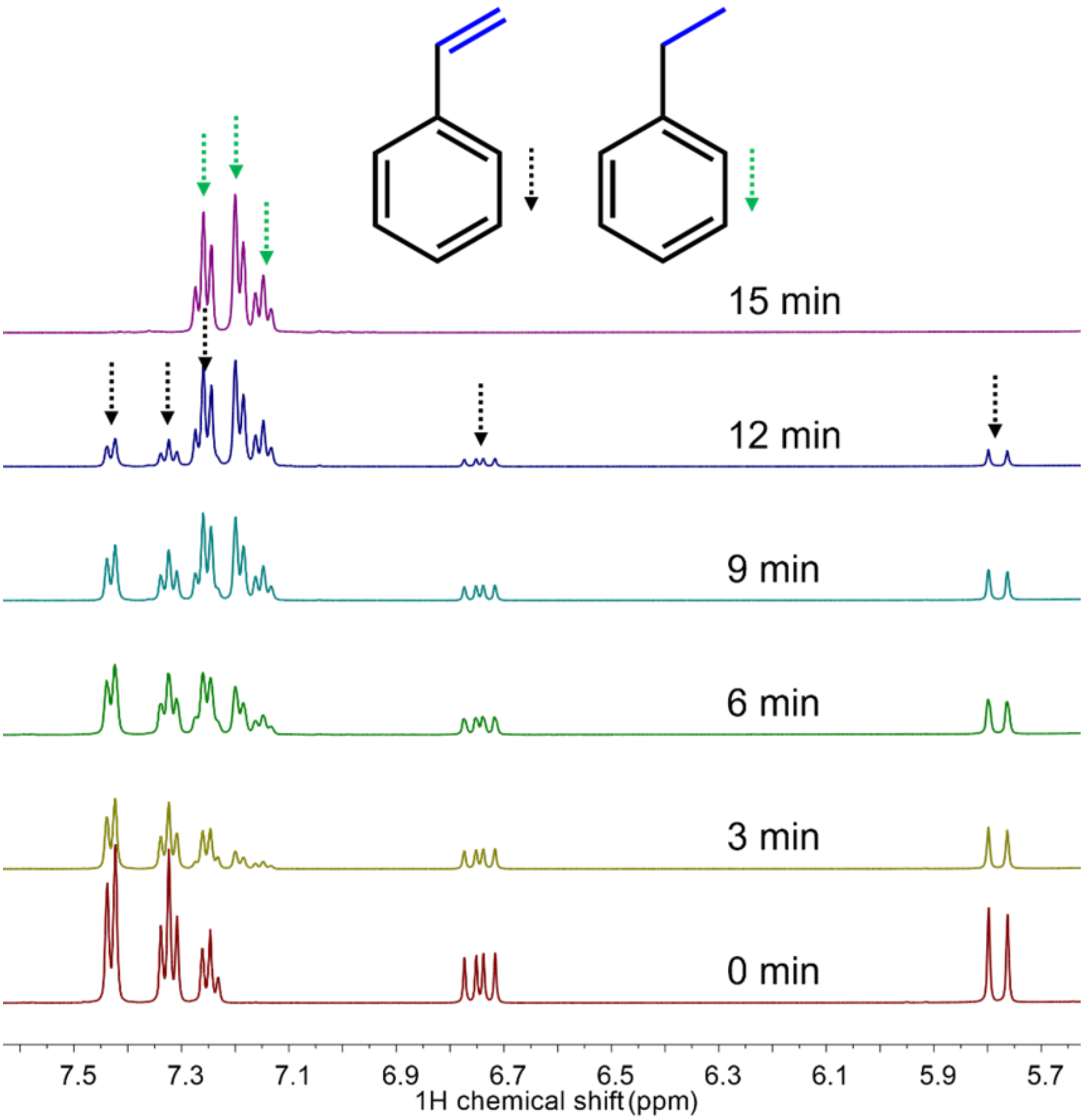

Figure S12. ^{1}H NMR spectra (in CD_3OD) of styrene hydrogenation over Pt NPs/C in EtOH at different time points. Black dotted and green dotted arrows indicate styrene and ethylbenzene, respectively.

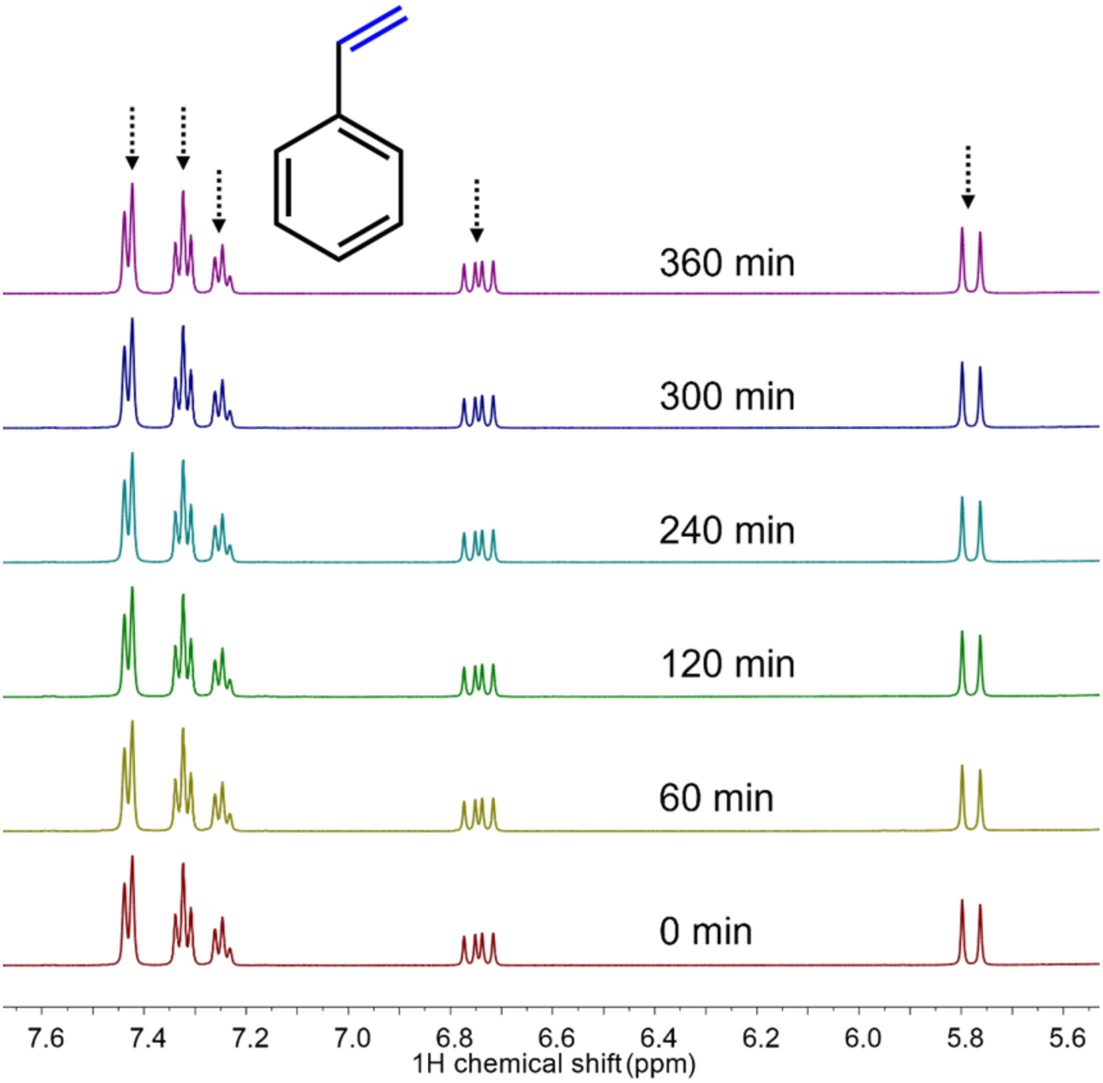

Figure S13. ^{1}H NMR spectra (in CD_3OD) of styrene hydrogenation over HPP-Pt NPs/C in EtOH at different time points. Black dotted arrows indicate styrene.

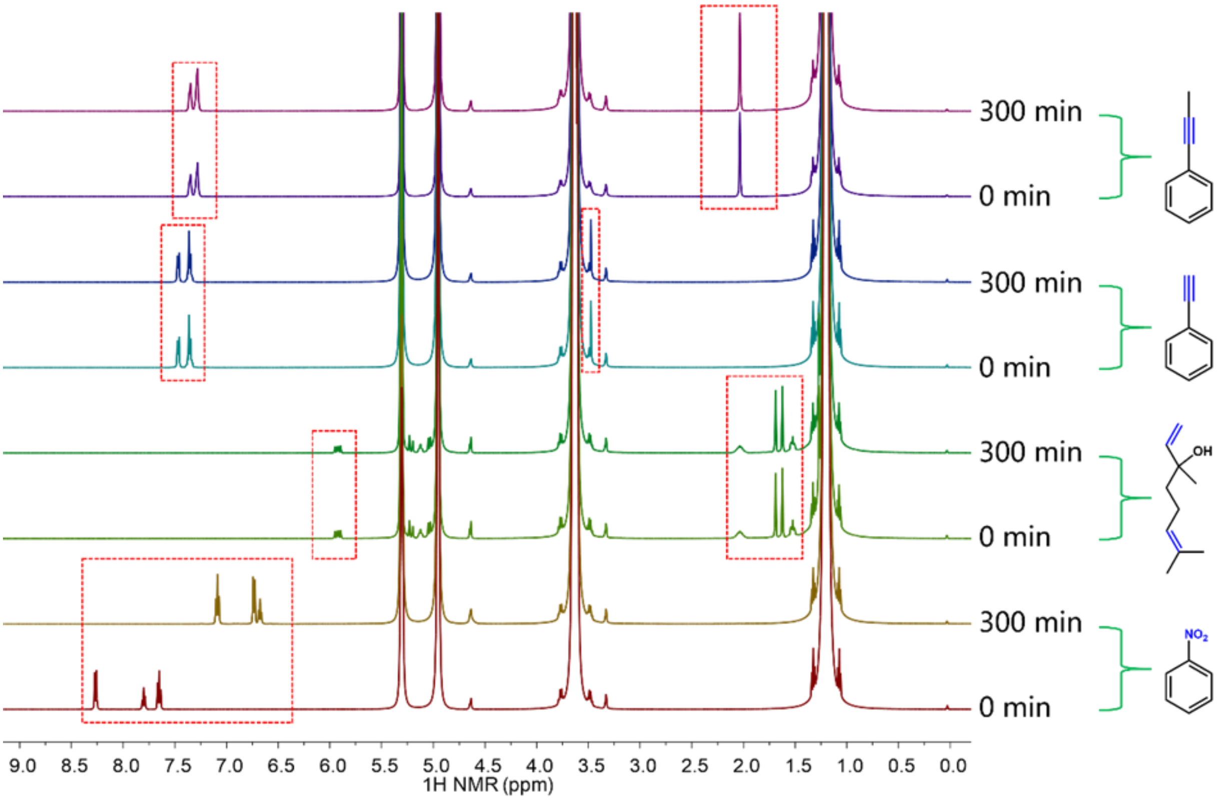

Figure S14. Oxygen-isolated ^{1}H NMR spectra (in CD_3OD) of NB, linalool, phenylacetylene and 1-phenyl-1-propyne hydrogenation over HPP-Pt NPs/C in EtOH at different time points. The conversions of linalool, phenylacetylene and 1-phenyl-1-propyne were nearly negligible under identical conditions as NB hydrogenation.

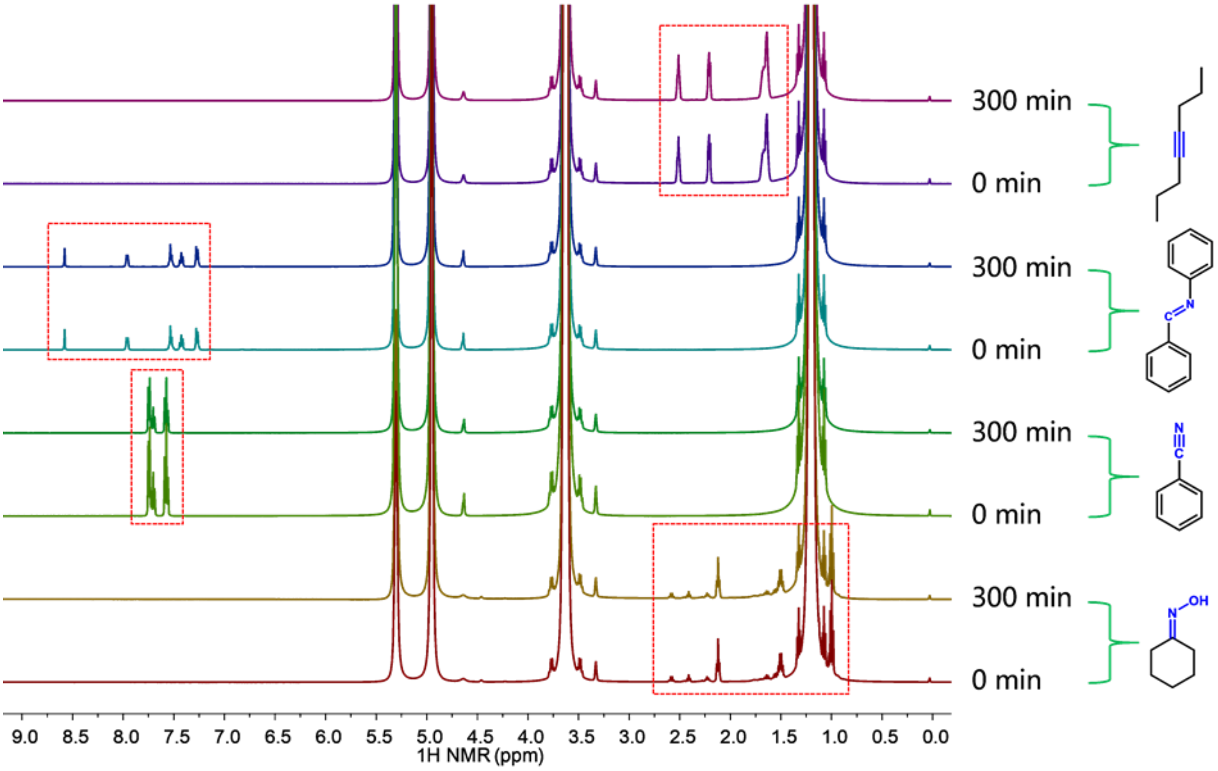

Figure S15. Oxygen-isolated ^{1}H NMR spectra (in CD_3OD) of cyclohexanone oxime, benzonitrile, benzylideneaniline and oct-4-yne hydrogenation over HPP-Pt NPs/C in EtOH at different time points. The conversions of cyclohexanone oxime, benzonitrile, benzylideneaniline and oct-4-yne were nearly negligible under identical conditions as NB hydrogenation.

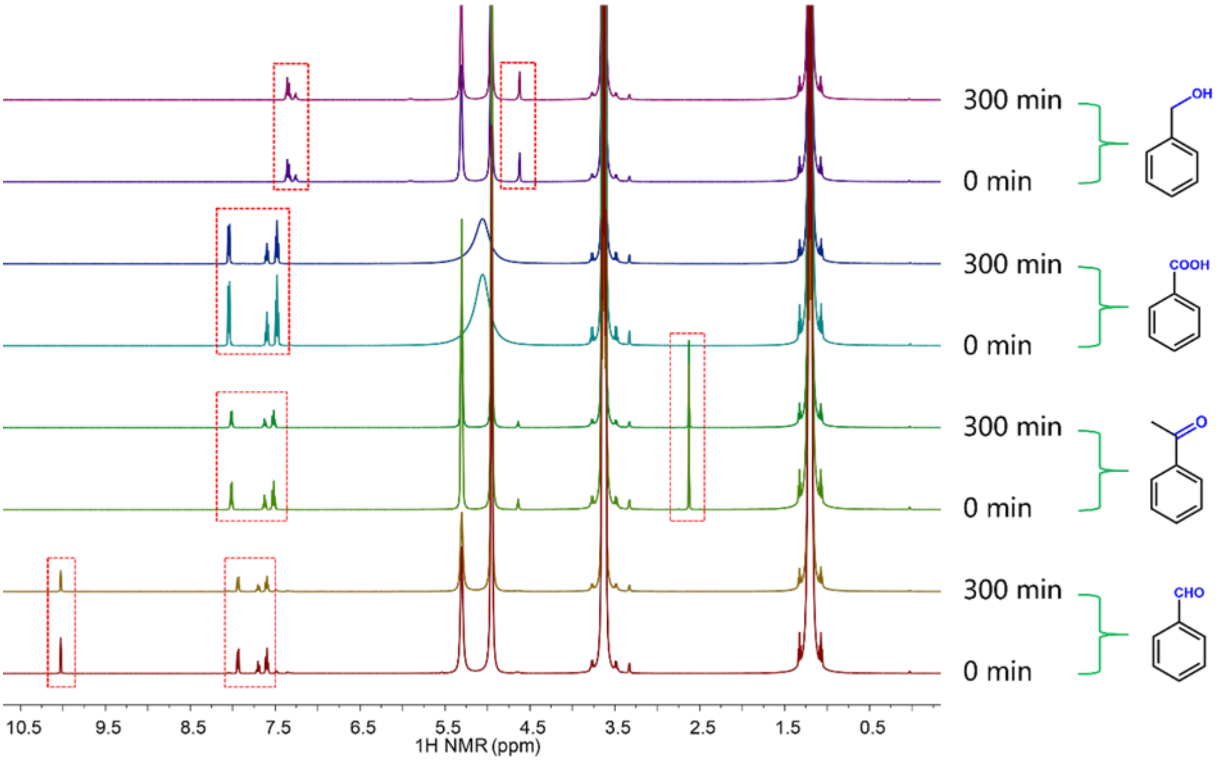

Figure S16. Oxygen-isolated ^{1}H NMR spectra (in CD_3OD) of benzaldehyde, acetophenone, benzoic acid and benzyl alcohol hydrogenation over HPP-Pt NPs/C in EtOH at different time points. The conversions of benzaldehyde, acetophenone, benzoic acid and benzyl alcohol were nearly negligible under identical conditions as NB hydrogenation.

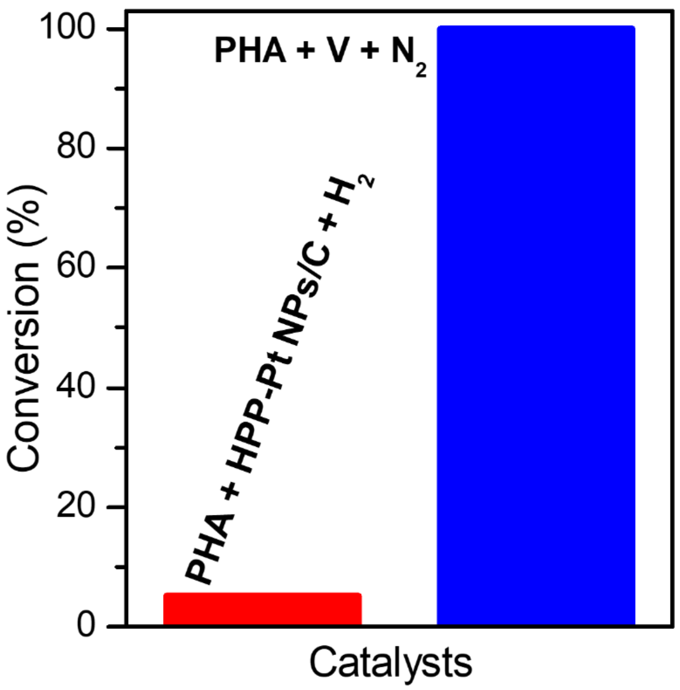

Figure S17. PHA hydrogenation catalyzed by HPP-Pt NPs/C (red bar) and PHA disproportionation facilitated by V [VO(acac)$_2$, blue bar] in EtOH under N_2. Reaction time: 40 min.

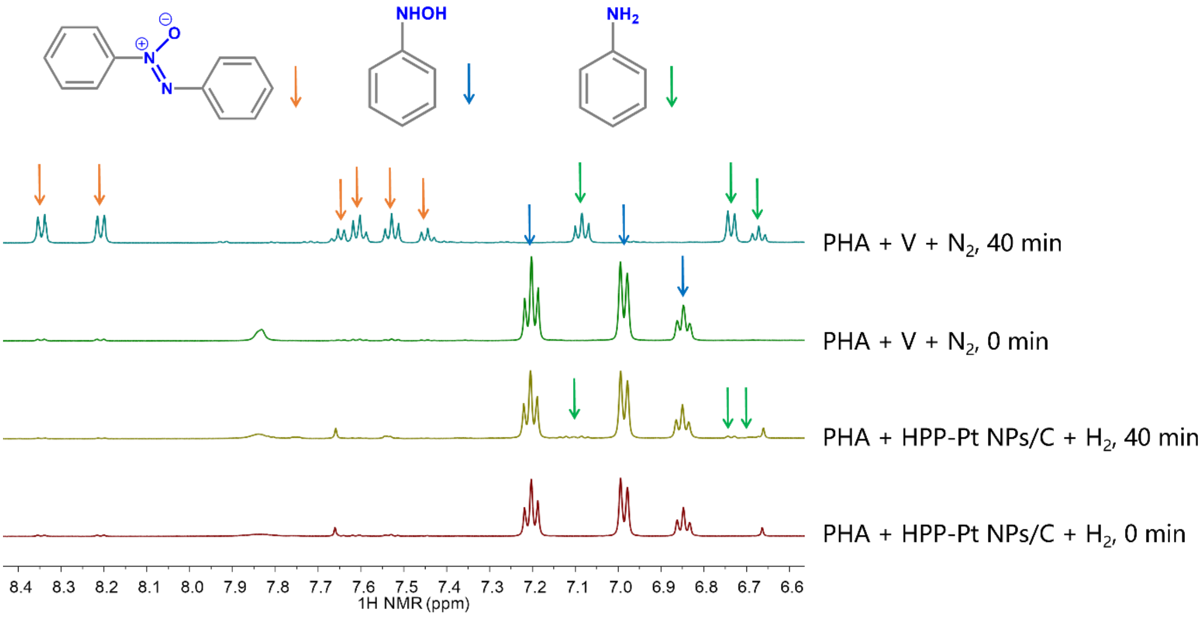

Figure S18. Oxygen-isolated ^{1}H NMR spectra (in CD_3OD) of PHA hydrogenation catalyzed by HPP-Pt NPs/C and PHA disproportionation facilitated by V [$VO(acac)_2$] in N_2. Blue, yellow and green arrows indicate PHA, AXB and AN, respectively.

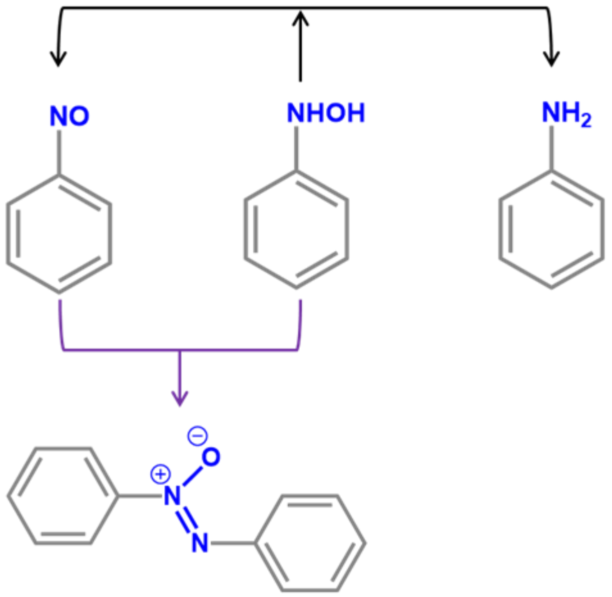

Figure S19. Illustration for disproportionation (black arrows) of PHA and condensation of PHA with NSB (purple arrow).

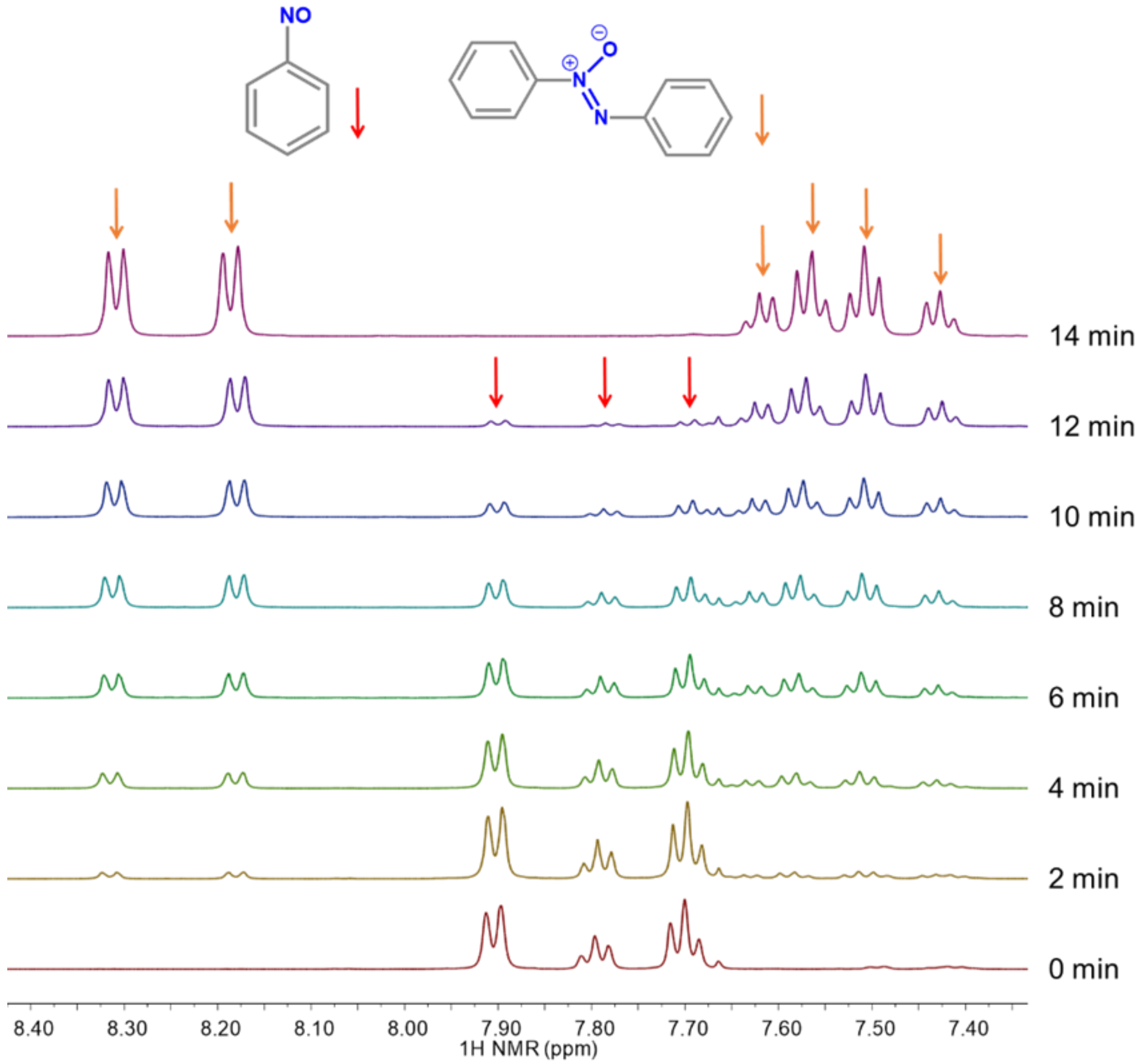

Figure S20. Oxygen-isolated ^{1}H NMR spectra (in CD_3OD) of NSB hydrogenation catalyzed by HPP-Pt NPs/C at different reaction times. Red and yellow arrows indicate NSB, and AXB, respectively.

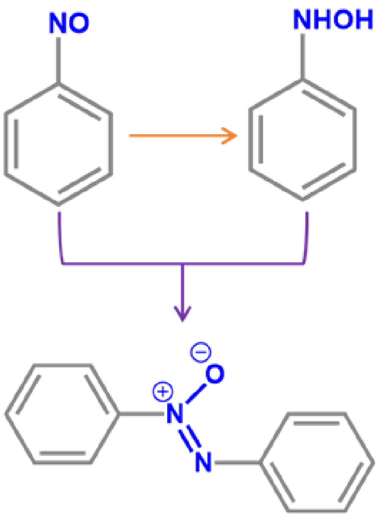

Figure S21. Illustration for hydrogenation (yellow arrow) of NSB and condensation of PHA with NSB (purple arrow).

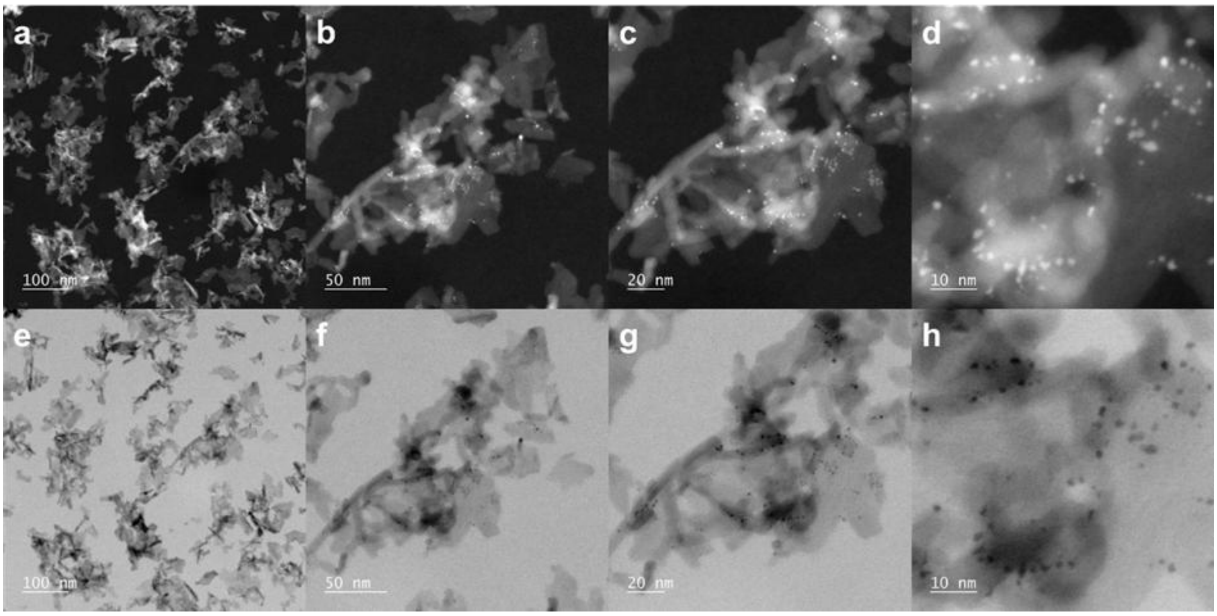

Figure S22. STEM (a-d) and TEM (e-h) images of HPP-Pt NPs/γ-Al_2O_3.

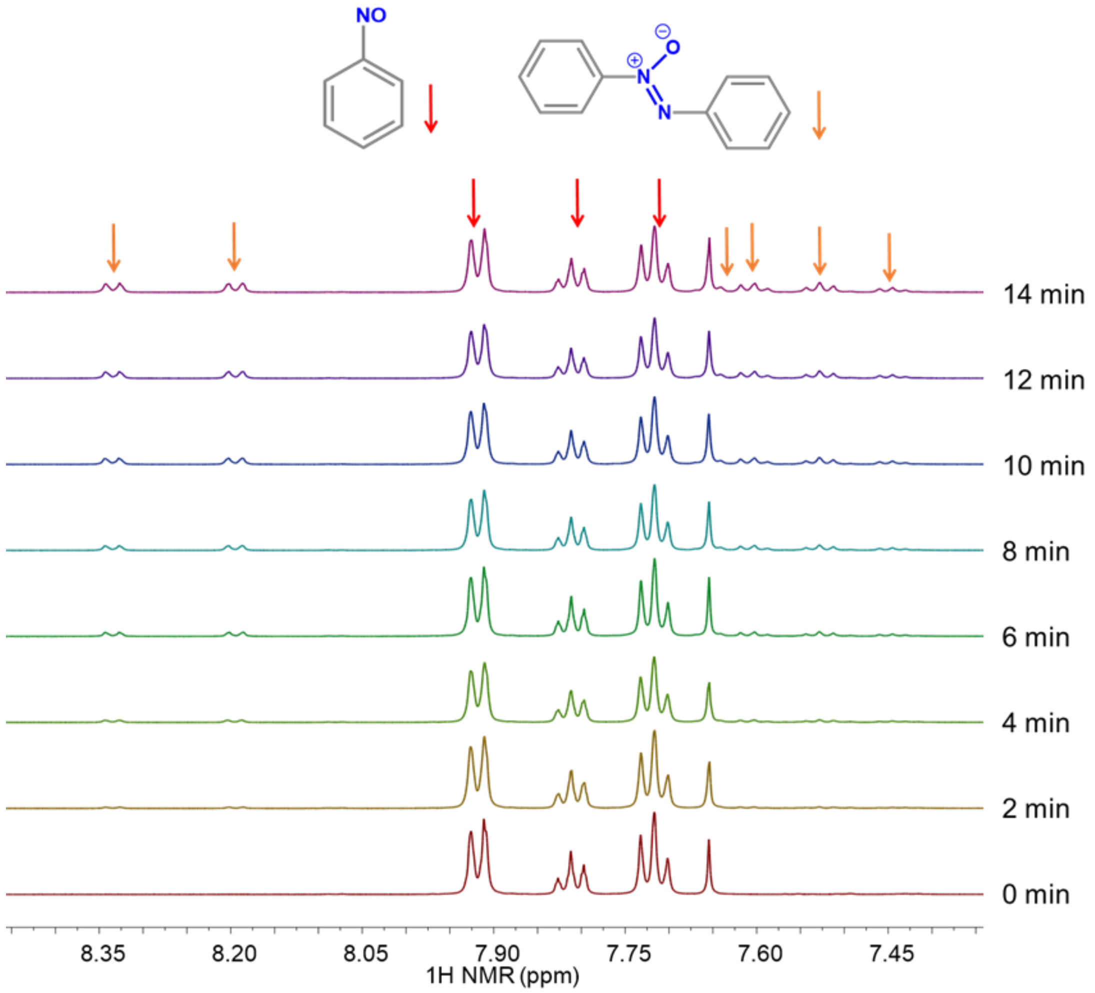

Figure S23. Oxygen-isolated ^{1}H NMR spectra (in CD_3OD) of NSB hydrogenation catalyzed by HPP-Pt NPs/γ-Al_2O_3 at different reaction times. Red and yellow arrows indicate NSB and AXB, respectively. Comparative kinetic analysis between HPP-Pt NPs/C (carbon + interface active sites) and HPP-Pt NPs/γ-Al_2O_3 (interface-only active sites) revealed electron transfer through carbon surfaces is ~6 times faster than through ligand/support-metal interfaces (Figures 4a, S20 for quantification methodology).

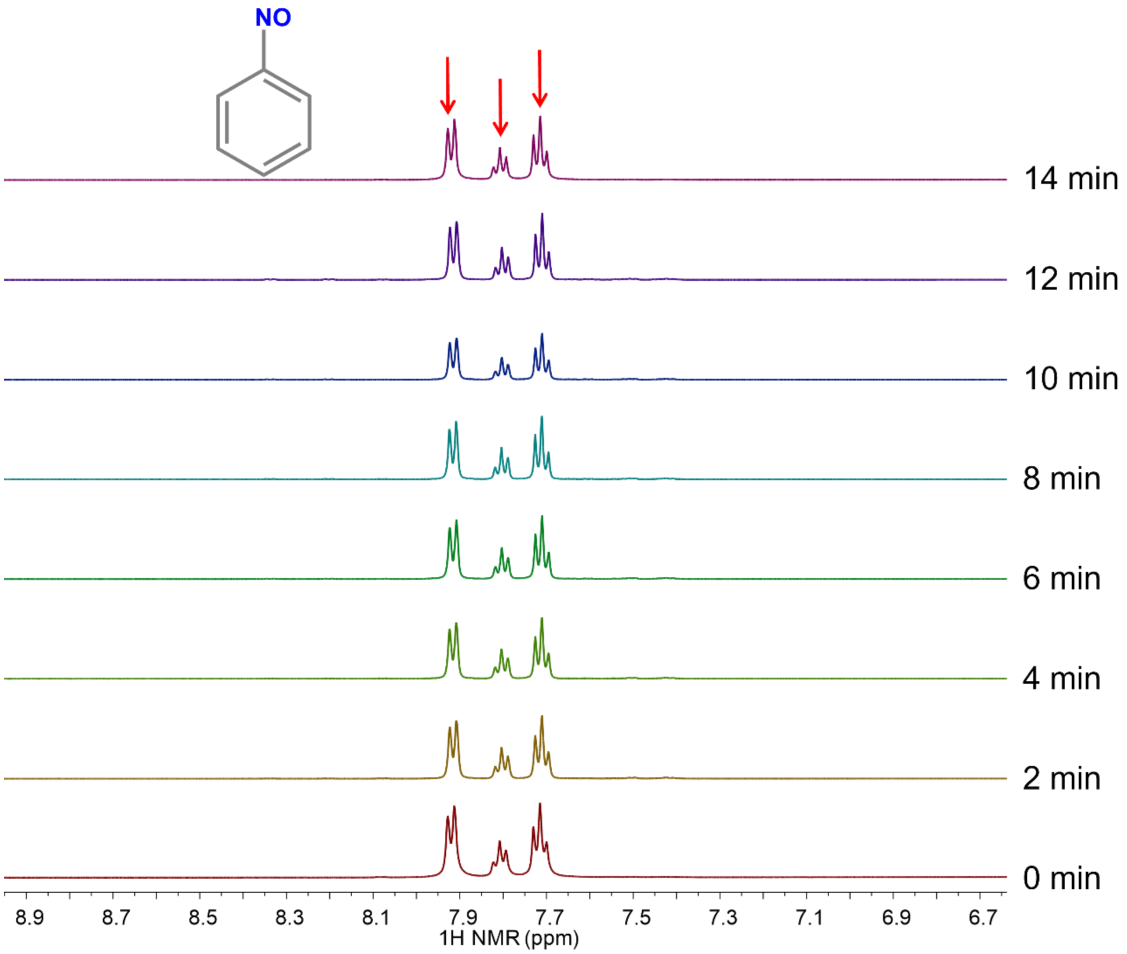

Figure S24. Oxygen-isolated ^{1}H NMR spectra (in CD_3OD) of NSB hydrogenation catalyzed by HPP-C at different reaction times. Red arrows indicate NSB.

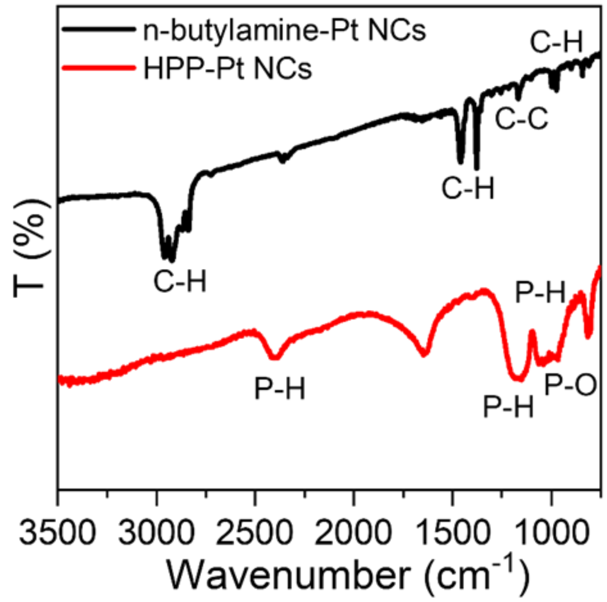

Figure S25. IR spectra of n-butylamine adsorption on Pt NCs and H_3PO_2 adsorption on Pt NCs (HPP-Pt NCs). After ligand exchange, the amine ligands were replaced by H_3PO_2.

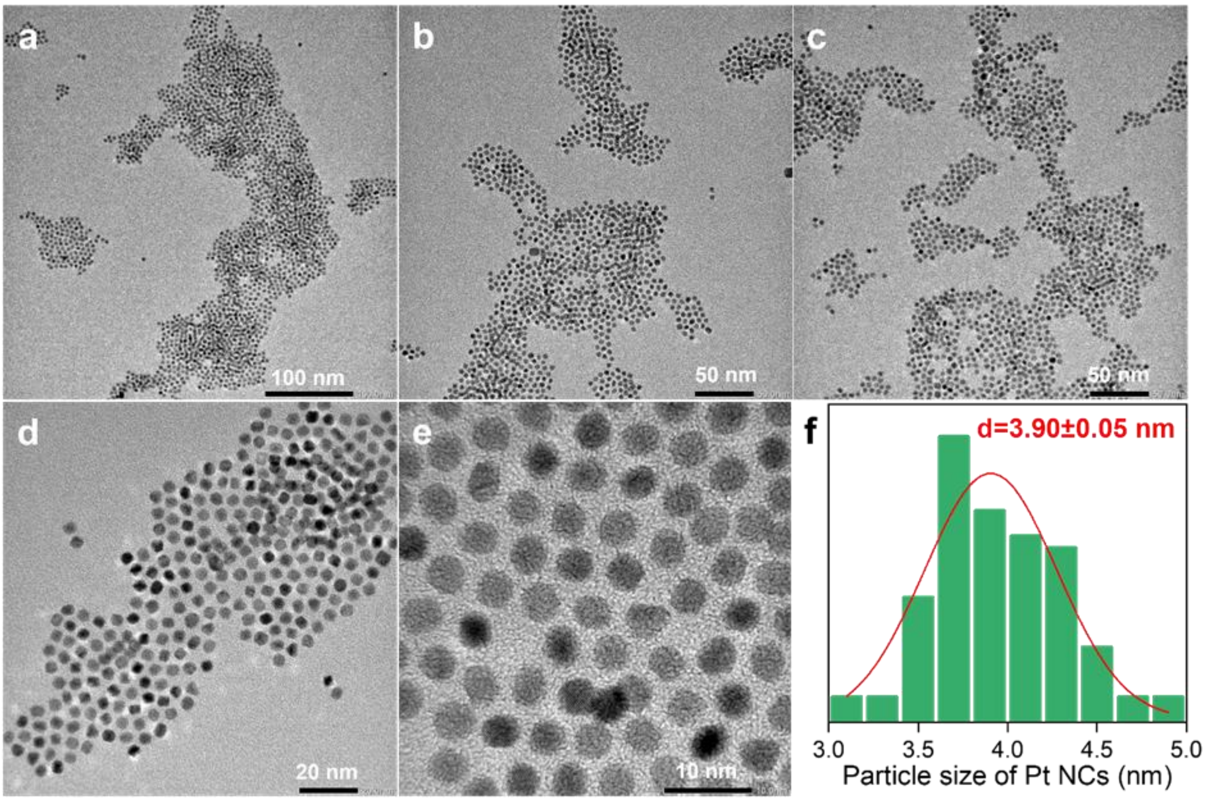

Figure S26. TEM images (a-e) and nanocrystal size distribution histogram (f) of HPP-Pt NCs. The monodisperse Pt NCs were synthesised successfully, exhibiting an average particle size of ~3.9 nm.

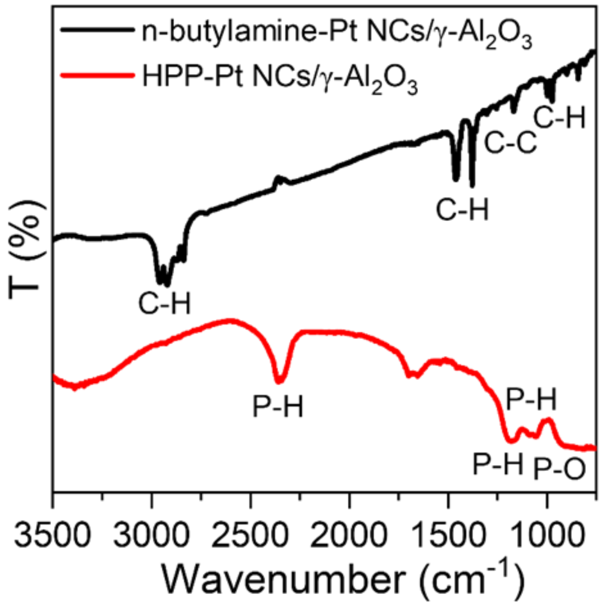

Figure S27. IR spectra of n-butylamine adsorption on Pt NCs/γ-Al_2O_3 and H_3PO_2 adsorption on Pt NCs/γ-Al_2O_3 (HPP-Pt NCs/γ-Al_2O_3). After thermal treatment and ligand exchange, the amine ligands are replaced by H_3PO_2.

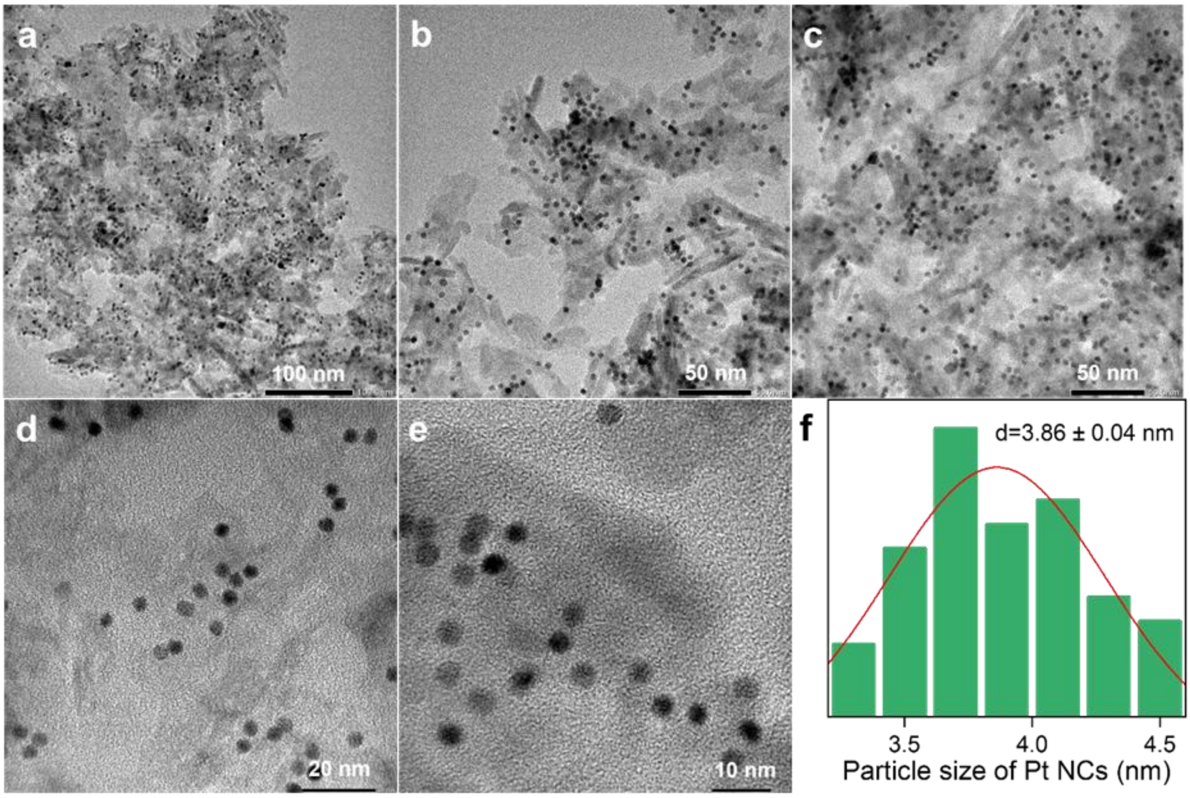

Figure S28. TEM images (a-e) and nanocrystal size distribution histogram (f) of HPP-Pt NCs/γ-Al_2O_3. The HPP-Pt NCs/γ-Al_2O_3 shows negligible change in the average particle size (~3.9 nm) of the Pt NCs.

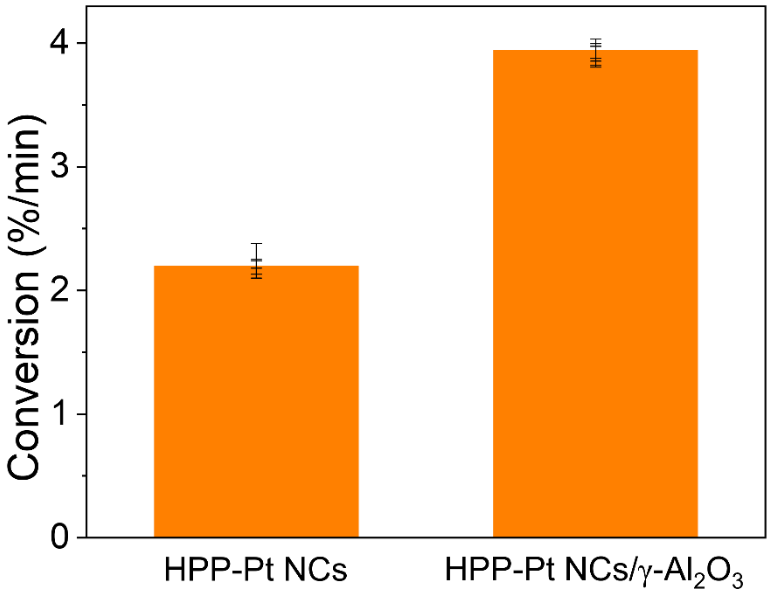

Figure S29. Comparative catalytic performance of HPP-Pt NCs and HPP-Pt NCs/γ-Al_2O_3 in NSB hydrogenation. The average conversion rates are 2.20 %/min for HPP-Pt NCs (ligand-metal interfaces only), and 3.90 %/min for HPP-Pt NCs/γ-Al_2O_3 (ligand + support-metal interfaces). The 1.70 %/min difference represents the metal-support interface contribution, indicating that the electron transfer efficiency at the ligand-metal interface is ~1.29 times higher than that at the metal-support interface.

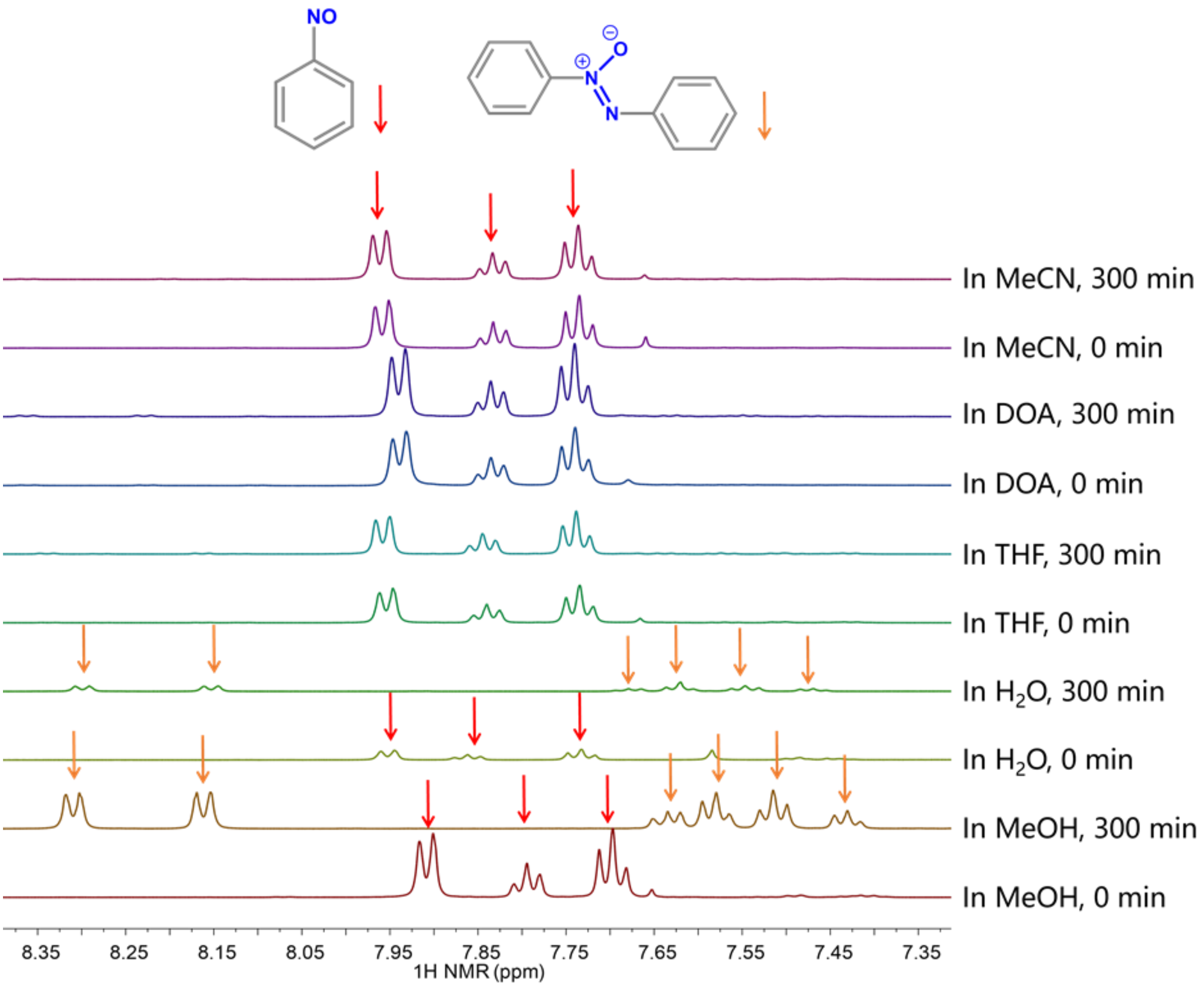

Figure S30. Oxygen-isolated ^{1}H NMR spectra (in CD_3OD) of NSB hydrogenation catalyzed by HPP-Pt NPs/C in different solvents. Red and yellow arrows indicate NSB and AXB, respectively.

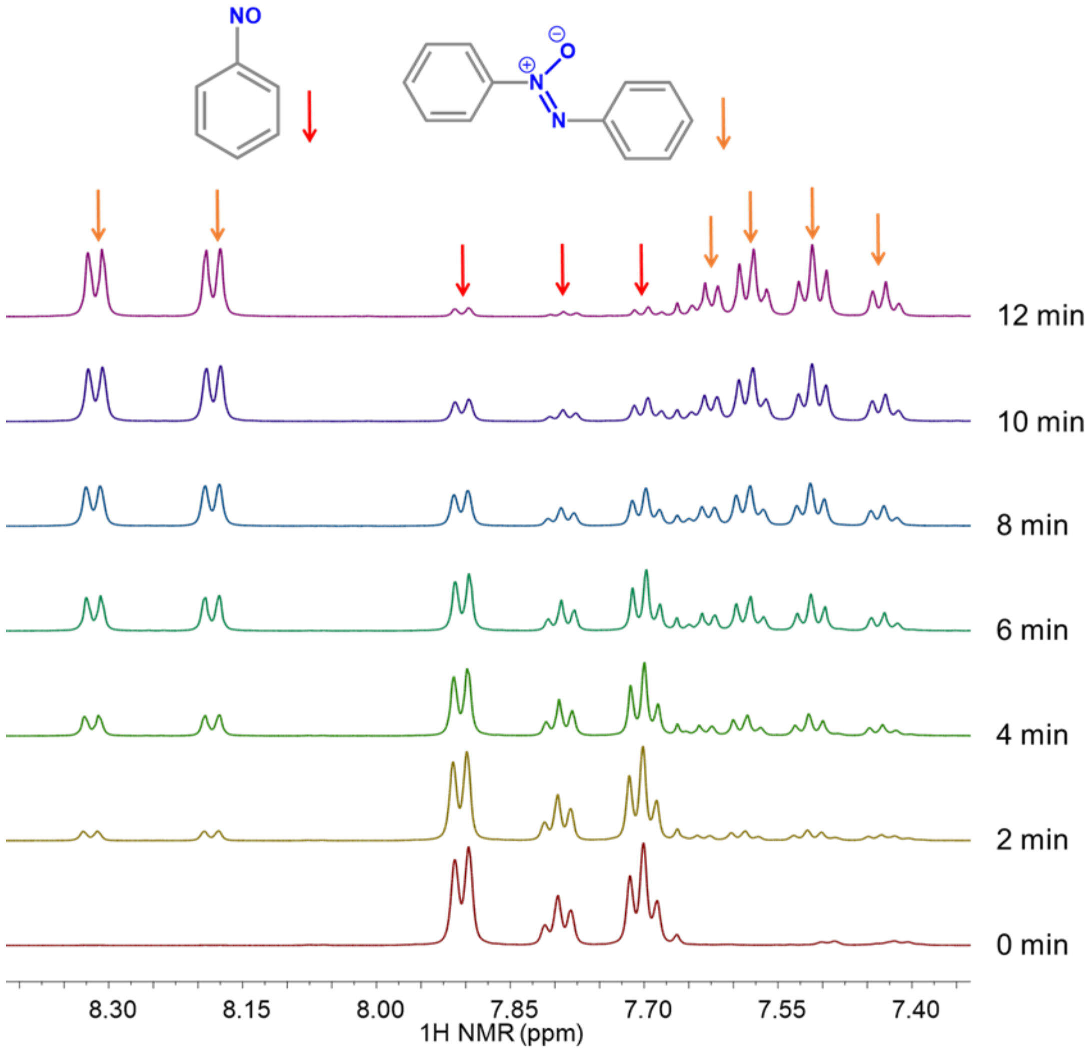

Figure S31. Oxygen-isolated ^{1}H NMR spectra (in CD_3OD) of NSB deuteration catalyzed by HPP-Pt NPs/C at different reaction times. Red and yellow arrows indicate NSB and AXB, respectively.

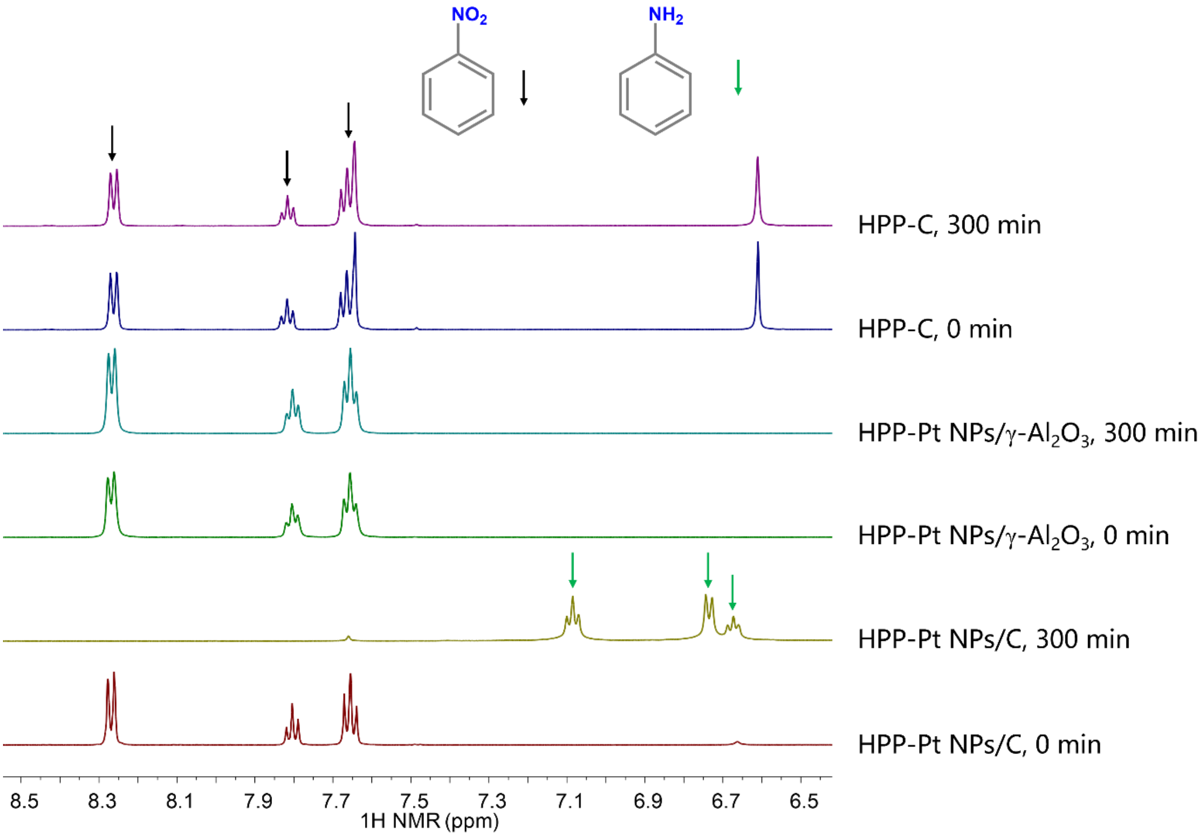

Figure S32. Oxygen-isolated ^{1}H NMR spectra (in CD_3OD) of NB hydrogenation catalyzed by HPP-Pt NPs/C, HPP-Pt NPs/γ-Al_2O_3 and HPP-C catalysts. Red and yellow arrows indicate NSB and AXB, respectively.

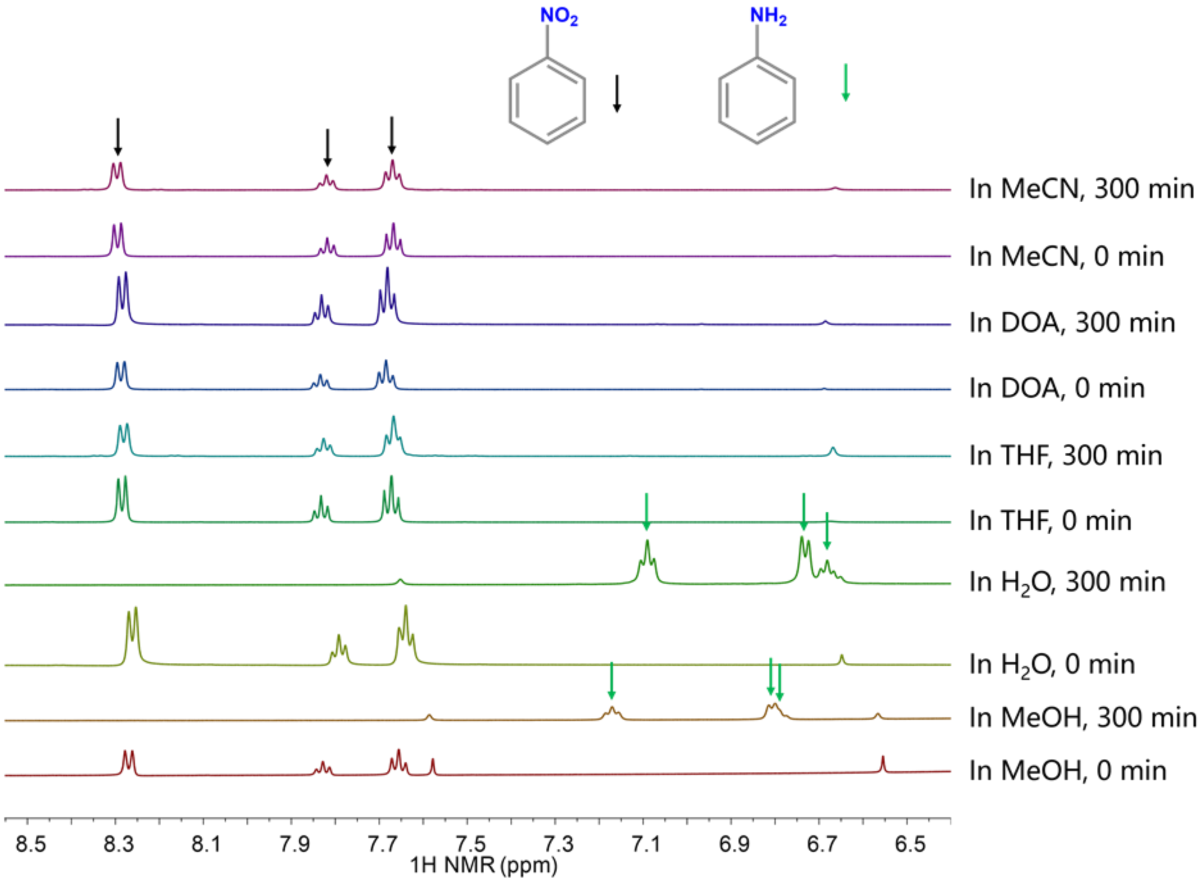

Figure S33. Oxygen-isolated ^{1}H NMR spectra (in CD_3OD) of NB hydrogenation catalyzed by HPP-Pt NPs/C in different solvents. Black and green arrows indicate NB and AN, respectively.

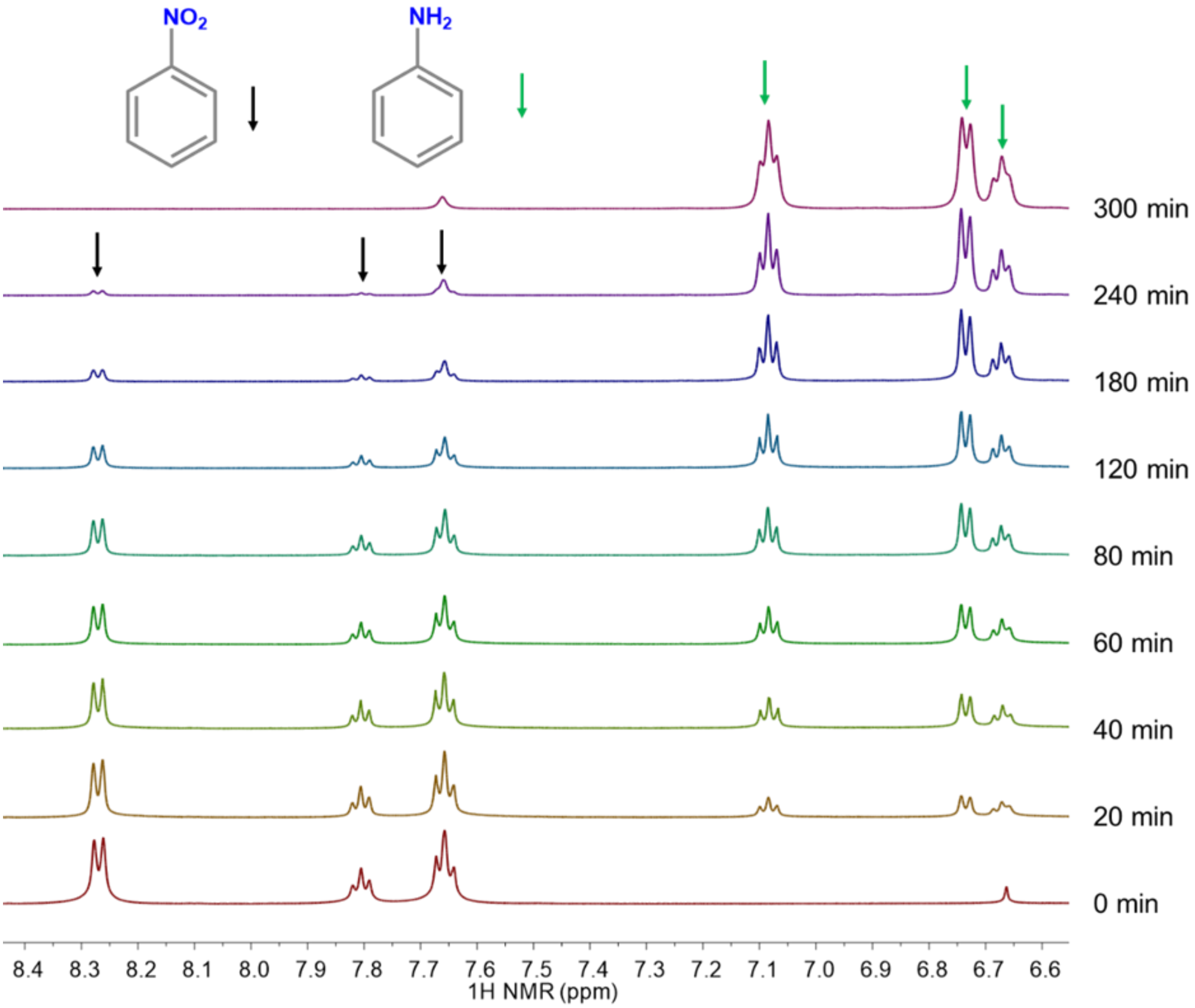

Figure S34. Oxygen-isolated ^{1}H NMR spectra (in CD_3OD) of NB hydrogenation catalyzed by HPP-Pt NPs/C. Black and green arrows indicate NB and AN, respectively.

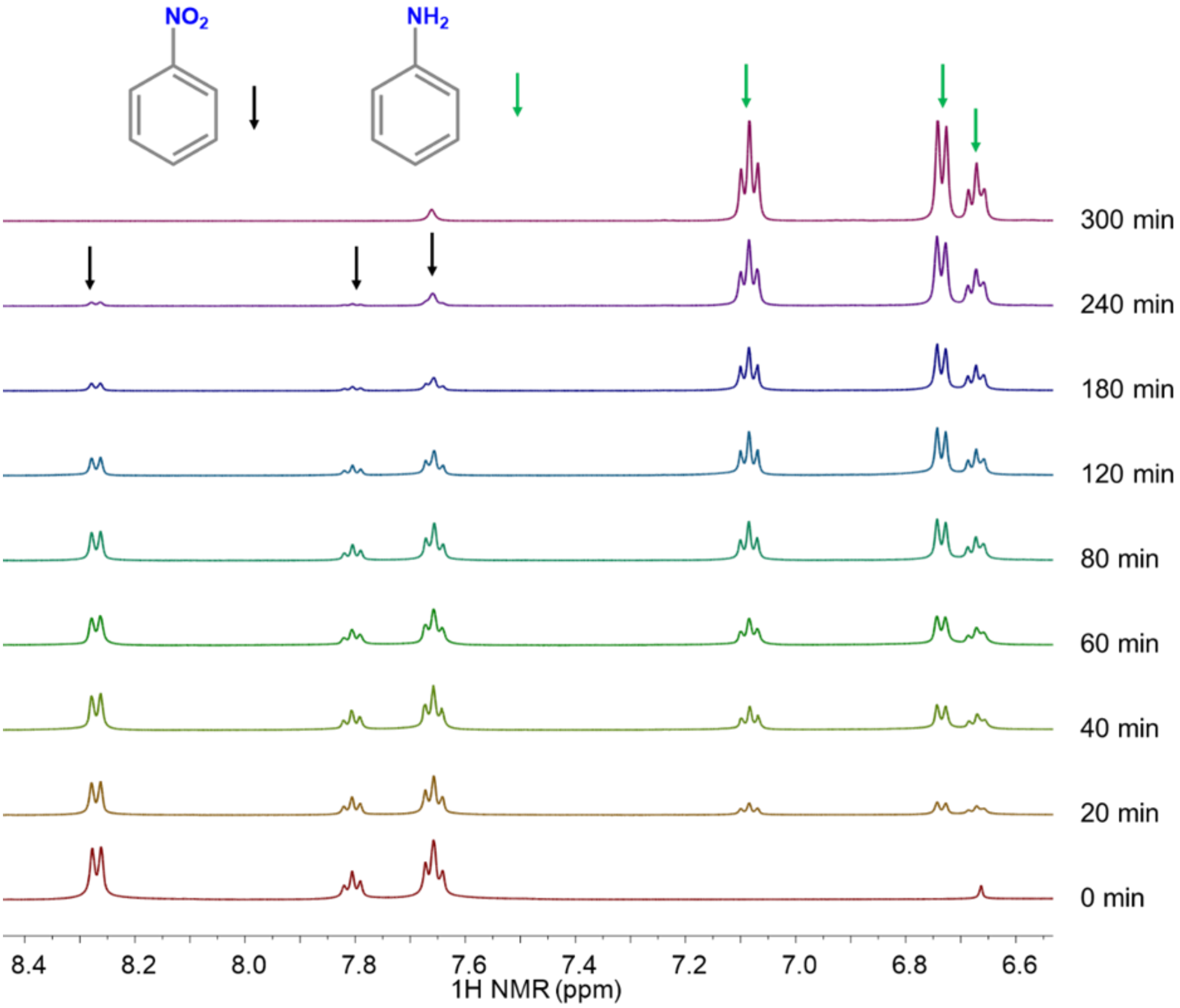

Figure S35. Oxygen-isolated ^{1}H NMR spectra (in CD_3OD) of NB deuteration catalyzed by HPP-Pt NPs/C. Black and green arrows indicate NB and AN, respectively.

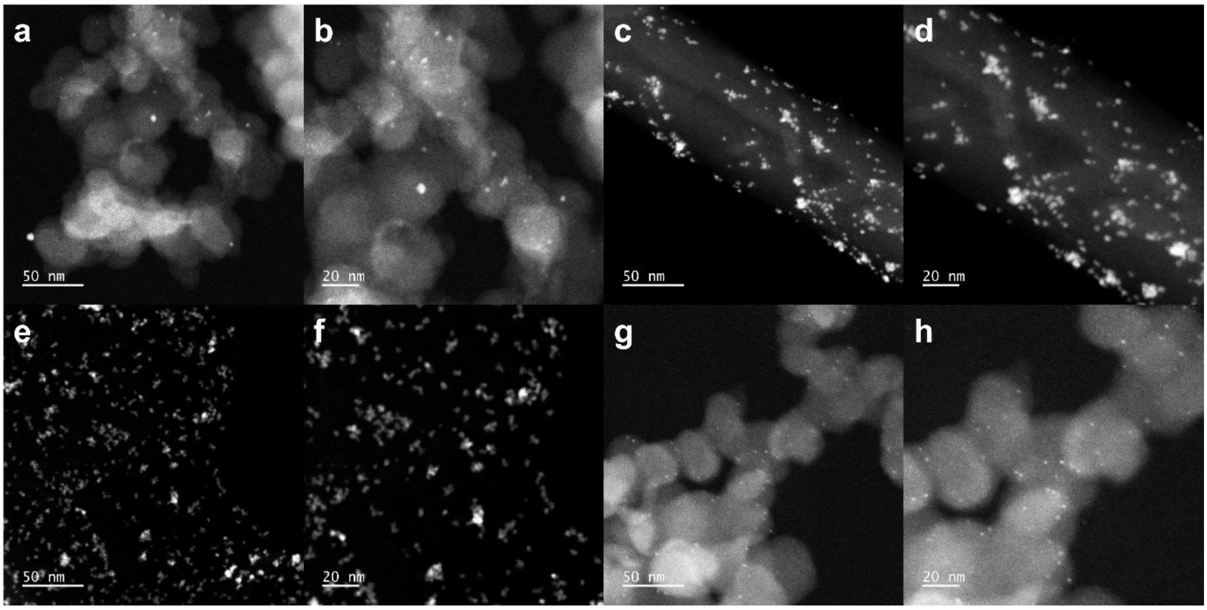

Figure S36. STEM images of H_3PO_2-modified Pd NPs/XC-72 (a, b), H_3PO_2-modified Pt NPs/CNT (c, d), H_3PO_2-modified Pt NPs/GR (e, f) and H_3PO_3-modified Pt NPs/C (g, h).

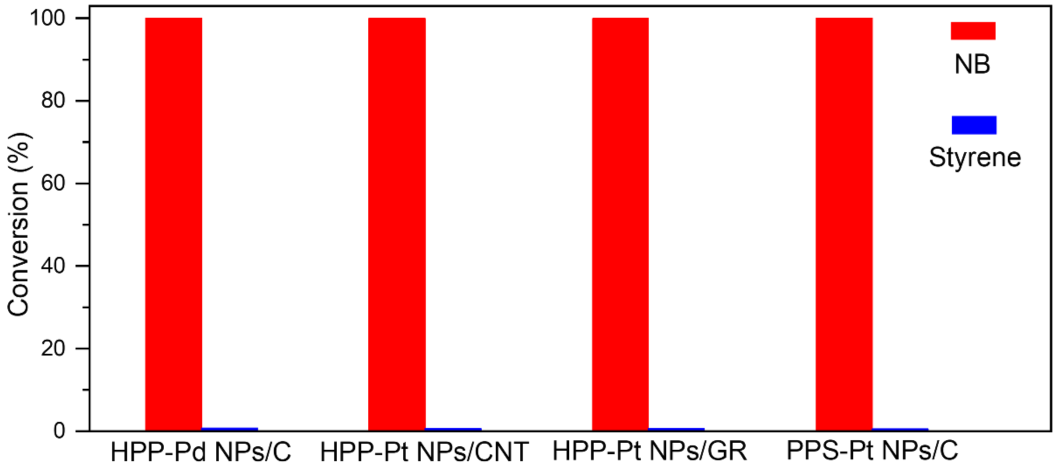

Figure S37. Catalytic performance of H_3PO_2-modified Pd NPs/XC-72 (HPP-Pd NPs/C), H_3PO_2-modified Pt NPs/CNT (HPP-Pt NPs/CNT), H_3PO_2-modified Pt NPs/GR (HPP-Pt NPs/GR) and H_3PO_3-modified Pt NPs/XC-72 (PPS-Pt NPs/C) for the hydrogenation of NB and styrene. Reaction time: 300 min.

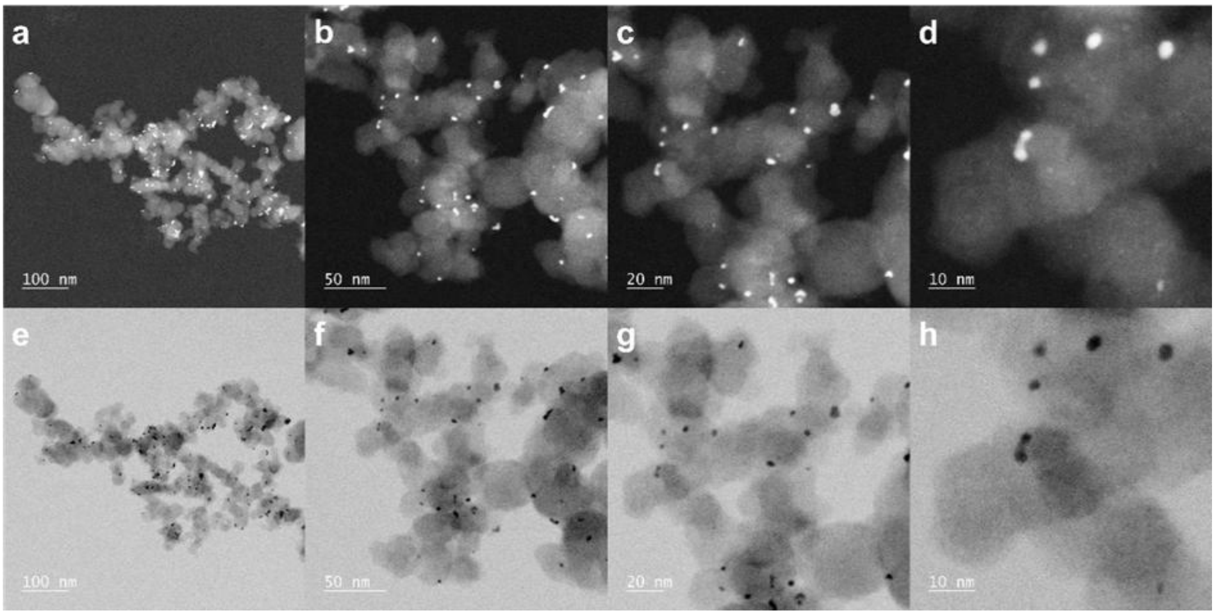

Figure S38. STEM (a-d) and TEM (e-h) images of H_3PO_2-modified commercial Pt NPs/C (2 wt%). Distinct Pt NPs are supported on the carbon support.

References

[1] Chen, G.; Yang, H.; Wu, B.; Zheng, Y.; Zheng, N. Supported Monodisperse Pt Nanoparticles from $[Pt_3(CO)_3(\mu_2\text{-}CO)_3]_5^{2-}$ Clusters for Investigating Support-Pt Interface Effect in Catalysis. 2013, Dalton Trans., 42, 12699, 10.1039/c3dt50942g

[2] Wu, Q.; Su, W.; Huang, R., et al. Full Selectivity Control over the Catalytic Hydrogenation of Nitroaromatics into Six Products. 2024, Angew. Chem. Int. Ed., 63, e202408731, 10.1002/anie.202408731

[3] Bekins, B. A.; Warren, E.; Godsy, E. M. A Comparison of Zero-Order, First-Order, and Monod Biotransformation Models. 1998, Groundwater, 36, 261, 10.1111/j.1745-6584.1998.tb01091.x
